# Soil microbial communities are sensitive to differences in fertilization intensity in organic and conventional farming systems

**DOI:** 10.1093/femsec/fiad046

**Published:** 2023-05-09

**Authors:** Martina Lori, Martin Hartmann, Dominika Kundel, Jochen Mayer, Ralf C Mueller, Paul Mäder, Hans-Martin Krause

**Affiliations:** Department of Soil Sciences, Research Institute of Organic Agriculture (FiBL), Ackerstrasse 113, 5070 Frick, Switzerland; Department of Environmental Systems Science, ETH Zürich, Universitätstrasse 2, 8092 Zürich, Switzerland; Department of Soil Sciences, Research Institute of Organic Agriculture (FiBL), Ackerstrasse 113, 5070 Frick, Switzerland; Agroecology and Environment, Agroscope, Reckenholzstrasse 191, 8046 Zürich, Switzerland; Agroecology and Environment, Agroscope, Reckenholzstrasse 191, 8046 Zürich, Switzerland; Department of Soil Sciences, Research Institute of Organic Agriculture (FiBL), Ackerstrasse 113, 5070 Frick, Switzerland; Department of Soil Sciences, Research Institute of Organic Agriculture (FiBL), Ackerstrasse 113, 5070 Frick, Switzerland

**Keywords:** DOK trial, farmyard manure, long-term field experiment, soil microbial diversity, soil quality, stocking density

## Abstract

Intensive agriculture has increased global food production, but also impaired ecosystem services and soil biodiversity. Organic fertilization, essential to organic and integrated farming, can provide numerous benefits for soil quality but also compromise the environment by polluting soils and producing greenhouse gases through animal husbandry. The need for reduced stocking density is inevitably accompanied by lower FYM inputs, but little research is available on the impact of these effects on the soil microbiome. We collected soil samples from winter wheat plots of a 42-year-old long-term trial comparing different farming systems receiving farmyard manure at two intensities and measured soil quality parameters and microbial community diversity through DNA metabarcoding. High-input fertilization, corresponding to 1.4 livestock units (LU) improved the soil’s nutritional status and increased soil microbial biomass and respiration when compared to low-input at 0.7 LU. Bacterial and fungal α-diversity was largely unaffected by fertilization intensity, whereas their community structure changed consistently, accompanied by an increase in the bacterial copiotroph-to-oligotroph ratio in high-input systems and by more copiotrophic indicator OTUs associated with high than low-input. This study shows that reduced nutrient availability under low-input selects oligotrophic microbes efficiently obtaining nutrients from various carbon sources; a potentially beneficial trait considering future agroecosystems.

## Teaser

Fertilization intensity shapes soil fungal and bacterial communities in organic and conventional farming systems of the 42-year-old DOK long-term field trial.

## Introduction

The green revolution substantially increased global food production, but also led to large-scale degradation of soil with severe consequences for human health and the environment (Gomiero et al. 2011, Fowler et al. 2013, Yang et al. 2020). There is an urgent need for more sustainable agroecosystems that do not compromise ecosystem health, but rather restore already degraded soils while providing sufficient yields to meet the growing global food demand. Microbes play a pivotal role in the functioning of agroecosystems (Wagg et al. 2014, Bender et al. 2016, Díaz-Rodríguez et al. 2021, Suman et al. 2022) and understanding the factors shaping soil microbial diversity is of prime interest to counteract soil degradation (Cavicchioli et al. 2019, Zhou et al. 2020).

Agricultural management factors such as tillage (Kraut-Cohen et al. 2020, Fang et al. 2022), fertilization (Wang et al. 2017, Bei et al. 2018), crop diversification (Stefan et al. 2021), and cover cropping (Kim et al. 2021) have shown to affect soil microbial communities. Farming systems (FS) implementing these practices into different management strategies have also been shown to promote distinct soil microbial communities (Hartmann et al. 2015, Bonanomi et al. 2016, Lupatini et al. 2017, Durrer et al. 2021).

Organic fertilization, a key element in organic farming, but also incorporated in conventional systems, is known to enhance soil quality by increasing soil organic carbon (SOC) content and improving soil structure compared to synthetic mineral nitrogen (N) fertilizer (Mäder et al. 2002, Liang et al. 2012, Francioli et al. 2016). Since most soil microbes obtain energy and nutrients for metabolic reactions from organic compounds, an increase in SOC is connected with stimulating effects on soil microbial biomass and activity (Ren et al. 2019, Ma et al. 2020a). A recent meta-analysis showed that replacing synthetic N with manure-based fertilizer at equivalent N rates improved crop productivity, reduced reactive N pollution, and increased SOC storage (Xia et al. 2017). Ren et al. (2019) identified in their meta-analysis that manure application increased soil microbial biomass carbon and nitrogen by around 40% and 55%, respectively, compared to mineral fertilized soils. Furthermore, a meta-analysis by Shu et al. (2022) synthesized that organic amendments significantly increased microbial diversity and shifted microbial community structure compared to mineral-only fertilization.

However, there are also concerning reports about the increased abundance of heavy metals, antibiotics, and pathogenic, even potentially antibiotic-resistant microorganisms, in soils receiving farmyard manure (FYM) (Kumar et al. 2013, Lin et al. 2016). Aside from possible soil pollution, animal husbandry itself strongly contributes to global warming through emissions of the greenhouse gases methane and nitrous oxide, calling for an urgent reduction of animal stocking densities (Eisen and Brown 2022).

For the above-mentioned reasons, it is crucial to understand how soil microbial communities react to long-term animal-manure-based fertilization at contrasting intensities. For example, a study by Wang et al. (2021) based on shotgun sequencing showed bacterial community structure as well as functional gene composition to differ across a gradient of organic fertilization in a grassland system whereas fungi and archaea, as well as microbial α-diversity, remained unaffected. A study on arable soils by Ma et al. (2020b) based on shotgun sequencing combined with stable isotope phosphorous (P) and carbon (C) labelling demonstrated that long-term FYM application alters soil organic P stocks and cycling and that microbial functional gene abundance was coupled with P cycling rates with differences between mean and high input systems.

To provide further knowledge about how animal-based manure fertilization intensity (FI) affects the soil microbiome, the present study aims to analyze how low-input compared to high-input fertilization affects bacteria and fungi in three FYM-receiving certified and practice-relevant FSs of the 42-year-old DOK [bioDynamic, bioOrganic, Konventionell (German for conventional)] arable long-term trial in Switzerland (Krause et al. 2020). For this purpose, we collected soil samples from a biodynamic (BIODYN), an organic (BIOORG) and a conventional (CONFYM) system, of which all received FYM at two fertilization intensities (FI; 0.7 livestock units (LU) and 1.4 LU). We then analyzed the nutritional status (C and N) of the soil, microbial biomass and respiration, and fungal and bacterial diversity.

Previous work of the DOK trial already identified the five FSs (additionally an unfertilized control (NOFERT) and an exclusively mineral fertilized system (CONMIN)) to harbor distinct microbial communities (Hartmann et al. 2015). The main factor distinguishing the systems was whether they received FYM, whereas the impact of plant protection regimes was of subordinate importance. As the FS effects are already well described (Hartmann et al. 2015. Esperschütz et al. 2007), we hereafter mainly focus on FI effects.

We hypothesized that the well-known nutritional constraints in the low-input systems of the DOK trial might select for more diverse and rather oligotrophic microbial communities being able to efficiently degrade more complex recalcitrant C compounds and likely retrieve energy and nutrients via different metabolic strategies compared to high-input systems. Therefore, we hypothesize to observe increased α-diversity under low-input systems accompanied by a shift in microbial community structure towards oligotrophic taxa based on previously suggested trophic modes (Fierer et al. 2007, Leff et al. 2015, Ho et al. 2017). Conversely, copiotrophic groups that preferentially metabolize labile C sources are expected to be enriched in high-input soils and CONFYM systems receiving the least recalcitrant organic inputs.

## Materials and methods

I. Study site description

The DOK long-term field experiment was established in 1978 and is located in Therwil, Switzerland (47°30′ N; 7°32′ E; 306 m above sea level). The soil is a haplic luvisol with 15% sand, 70% silt, and 15% clay and the climate is mild with a mean annual temperature of 10.5°C and mean annual precipitation of 842 mm (Krause et al. 2020). The trial compares four different FSs, of which two organic systems (BIODYN, BIOORG) and one conventional (CONFYM) system at two FI (0.7 and 1.4 LU) were included in the current study. The DOK experiment is based on a 7-year crop rotation (sixth crop rotation period: maize, soya, winter wheat, catch crop, potatoes, winter wheat, grass clover, and grass clover) in three temporally shifted parallels. The field plots are arranged as randomized split–block design with four replicates of each treatment and crop. A scheme of the experimental design layout is presented in Figure S1 (Supporting Information).

The high-input versions of BIODYN, BIOORG, and CONFYM receive organic fertilizer corresponding to 1.4 LU per hectare, while the low-input versions receive 0.7 LU per hectare. Aside from slurry, CONFYM receives stacked manure, BIOORG rotten manure, and BIODYN composted manure. The CONFYM system is additionally complemented with mineral fertilizer following Swiss fertilization recommendations (https://www.agrocontrol.ch/oln) and is, therefore, the system receiving the highest N inputs. Detailed information on annual nutrient inputs is provided in Table 1. During the first two crop rotation periods the manure-receiving systems were maintained at 0.6 and 1.2 LU, and from the third onwards at 0.7 and 1.4 LU, respectively, due to increasing stocking densities in the regionally applied FSs. Weed management in the BIOORG and BIODYN is done mechanically while in the CONFYM systems weeds are controlled with herbicides. Pests and diseases in the CONFYM system are controlled with synthetic insecticides and fungicides while in the BIODYN and BIOORG systems, only biological pest control agents are applied. In the BIODYN system biodynamic preparations are applied to soils, plants, and compost, and plant growth regulators in the CONFYM system. More detailed information about fertilizer treatment and management can be found in Table 1 and Krause et al. (2020).

II. Soil sampling

**Table 1.** Main characteristics and mean annual inputs of the different FSs in the DOK trial. The biodynamic (BIODYN), bioorganic (BIOORG), and conventional (CONFYM) FSs are maintained at two FI: 0.7 and 1.4 LU fertilization equivalents. LU numbers refer to the organic matter (OM) input before system-specific processing of manure inputs took place. Ntot refers to total nitrogen inputs; Nmin is the sum of NH_4_^+^-N and NO_3_−N from slurry or mineral fertilizer inputs. P refers to phosphorus. Data about mean annual inputs are retrieved from Krause et al. (2022).

	BIODYN		BIOORG		CONFYM	
Manure type	Composted FYM and slurry		Rotted FYM and slurry, small amounts of potassium magnesia		Stacked FYM and slurry	
Mineral fertilizer	–		–		Mineral N, P, K fertilizer according to federal guidelines	
Weed control	Mechanical		Mechanical		Mechanical and herbicides	
Disease control	Indirect		Indirect, copper		Fungicides	
Pest control	Plant extracts, biocontrol		Plant extracts, biocontrol		Insecticides	
Special treatments	Biodynamic preparations		–		Plant growth regulators	
	**0.7 LU**	**1.4 LU**	**0.7 LU**	**1.4 LU**	**0.7 LU**	**1.4 LU**
	(kg ha^−1^ yr^−1^)	(kg ha^−1^ yr^−1^)	(kg ha^−1^ yr^−1^)	(kg ha ^−1^ yr^−1^)	(kg ha^−1^ yr^−1^)	(kg ha^−1^ yr^−1^)
Ntot	47	93	48	96	86	171
Nmin	13	26	15	30	57	113
P	12	24	12	24	19	37
OM	956	1911	1016	2032	1157	2314

We sampled bulk soil between rows of winter wheat in late February 2019 before the first spring fertilization. For each plot (5 m × 20 m), 12 soil cores were taken with an auger to a depth of 20 cm (plough horizon), pooled, and immediately cooled. A total of 24 samples were taken [six treatment combinations (three FSs and two FI) from four field replicates]. The soil was immediately transported to the laboratory and sieved to 5 mm. Subsamples for microbial biomass and activity analyses were immediately stored at 4°C whereas subsamples for molecular analysis were stored at −20°C, and subsamples for soil chemical analyses were air-dried and stored at 4°C until further processing.

III. Biogeochemical analysis

To determine total soil nitrogen (N_tot_) and carbon content (C_org_), a subsample of the air-dried soil was ground, homogenized, and analyzed via dry combustion on a CN analyzer in duplicates (Elementar Analysensysteme GmbH, Vario MAX Cube, Hau, Germany). Soil pH was determined in an aqueous suspension 1:2.5 (weight/volumne). Permanganate oxidizable carbon (PoxC) was extracted and analyzed following the principle of Weil et al. (2003) and modified as described in Bongiorno et al. (2019). Microbial biomass C (C_mic_) and microbial biomass N (N_mic_) were assessed in triplicates using the chloroform fumigation method (Vance et al. 1987).

To determine soil basal respiration, subsamples of field-moist soil were incubated at 25°C for 7 days. Afterwards, soil basal respiration was assessed in triplicates by incubation of soil samples for 28 days in hermetically sealed microcosms, and the capture of CO_2_ in alkali acid traps (0.025 M NaOH) as described in von Arb et al. (2020). Soil basal respiration was defined as the average C-mineralization rate during the second week of incubation.

IV. Molecular biological analysis

For metabarcoding, DNA was extracted from lyophilized soil samples (400 mg) using the NucleoSpin 96 Soil kit (Machery-Nagel, Dürren, Germany) with the SL2 + Enhancer SX lysis buffer according to the manufacturer’s instructions. Each sample was extracted in two technical replicates and pooled afterwards. Before PCR, DNA was diluted (1:10) to reduce inhibitory effects on PCR. A two-step PCR approach using CS1/CS2-tagged (Fluidigm, South San Francisco, CA, USA) primers targeting the V3–V4 region of the 16S rRNA gene (341F and 806R as modified in Frey et al. 2016), and primers targeting the fungal internal transcribed spacer region ITS2 (ITS3ngsmix1-5 and ITS4ngsUni; Tedersoo and Lindahl 2016) was deployed. A negative control containing double-distilled water instead of DNA and positive controls containing ZymoBIOMICS Microbial Community DNA Standard (Zymo Research Corporation, Irvine, CA, USA) or a fungal mock community (Bakker 2018) were included, respectively. The first PCR with the CS1/CS2-tagged primers (Fluidigm) was performed in technical triplicates (Table S1, Supporting Information, for primer sequences and PCR cycling conditions) using an SYBR green approach (Kapa SYBR Fast qPCR Kit Master Mix (2×) Universal; Kapa Biosystems, Wilmington, MA, USA) on a CFX96 Touch Real-Time PCR Detection System (Bio-Rad Laboratories, Hercules, CA, USA). Subsequently, PCR triplicates were pooled and purified using a magnetic bead solution (https://openwetware.org/wiki/SPRI_bead_mix). A subsample of each purified DNA sample was loaded on an agarose gel (1.25%) for visualization and validation. The second PCR, library preparation, and paired-end sequencing on an Illumina MiSeq sequencing platform (Illumina, San Diego, CA, USA) using the MiSeq v3 chemistry were performed at the Genome Quebec Innovation Center (Montreal, Canada) according to the amplicon guidelines provided by Illumina. Raw sequences and meta-data are deposited in NCBI Sequence Read Archive (SRA) under the accession number PRJNA841851.

V. Bioinformatics

Raw sequence data were processed using the Euler Scientific Compute Cluster at ETH Zürich. For most steps, USEARCH v11.0.667 (Edgar 2010) commands were used with default settings unless stated otherwise.

### 16S rRNA gene V3–V4 amplicon sequencing

A total of 2 074 922 raw paired-end reads were obtained with a mean of 61 027 (SD 14 414) across all samples. The positive control sequence of bacteriophage phi X 174 and low-complexity reads were removed with FILTER_PHIX and FILTER_LOWC, respectively. The remaining reads were trimmed with FASTX_TRUNCATE (stripleft 35, stripright 70), merged with FASTQ_MERGEPAIRS (fastq_minovlen 20, fastq_pctid 50, and fastq_minmergelen 100), and primer sequences searched and removed with SEARCH_PCR (minamp 200, maxamp 600). Reads were then quality filtered using PRINSEQ v0.20.4 (Morgulis et al. 2006) (rangelen 200–550, range-gc 30–70, min_qual_mean 20, ns_max_n 0, lc_thrshold 30) and dereplicated with FASTX_UNIQUES. Preprocessing resulted in 1 553 769 clean reads (mean 45 699, SD 10 687) with a mean length of 418 bp (SD 2.2).

### ITS2 amplicon sequencing

A total of 2 408 014 raw paired-end reads were obtained with a mean of 70 823 (SD 22 538) across all samples. The positive control sequence of bacteriophage phi X 174 and low-complexity reads were removed with FILTER_PHIX and FILTER_LOWC, respectively. The remaining reads were trimmed with FASTX_TRUNCATE (stripleft 45, stripright 85), merged with FASTQ_MERGEPAIRS (fastq_minovlen 20, fastq_pctid 60, and fastq_minmergelen 100), and primer sequences searched and removed with SEARCH_PCR (minamp 100, maxamp 600). Reads were then quality filtered using PRINSEQ v0.20.4 (rangelen 150–550, range-gc 30–70, min_qual_mean 20, ns_max_n 0, lc_thrshold 30), and dereplicated with FASTX_UNIQUES. Preprocessing resulted in 1 625 223 clean reads (mean 47 801, SD 15 254) with a mean length of 335 bp (SD 6.6).

### Clustering and taxonomy

The preprocessed 16S rRNA and ITS reads were clustered using CLUSTER_OTUS (Edgar 2013). The taxonomy for 16S and ITS data was assigned using SINTAX (Edgar 2016) (sintax_cutoff 0.85) based on the references SILVA_128_16S_utax_work.fa (Quast et al. 2013) and UNITE_v82_Fungi_04.02.2020.fasta, respectively (Abarenkov et al. 2010). Fungal annotation was additionally confirmed with ITSx (Bengtsson-Palme et al. 2013). Finally, 1 173 545 bacterial reads clustered into 7216 OTUs, and 1 552 348 fungal reads into 2881 OTUs.

Before downstream analysis nonbacterial (archaea, mitochondria, and chloroplasts) and nonfungal sequences were removed, resulting in 833 054 bacterial sequences assigned to 7114 OTUS, and 750 486 fungal sequences assigned to 2088 OTUs. Results of positive controls containing bacterial and fungal mock communities are shown in Figure S2 (Supporting Information).

VI. Statistical analysis

All analyses were conducted in r v 4.1.2 (R Core Team 2021) and r studio v 1.4.1717 (RStudio Team 2020) and plots were created using ggplot2 (Wickham 2016) unless indicated otherwise.

### Univariate datasets

Linear mixed-effect models using the nlme:: lme function (Pinheiro et al. 2020) were used to assess the effects of FI, FS, and their interaction on soil geochemical parameters, and microbial α-diversity. Field-plot nested in block was set as a random factor while varIdent modelled the inconsistent variance between FSs. The nlme:: anovoa_lme function (Pinheiro et al. 2020) was used to retrieve the statistical significance of the main effects. Estimated marginal means and 95% confidence intervals for each FI level within FSs were obtained using the emmeans:: emmeans function (Russell et al. 2020) and plotted along with the raw data. Planned contrasts between FI within FSs were again analyzed with the emmeans function if a significant main effect for FI or interaction effect has been found. Planned contrasts between FSs within FI levels were similarly analyzed if a significant FS or interaction effect has been found but results, hereof, are only provided in the supplementary information. Data were transformed using log, square-root, or inverse functions to satisfy the assumption of normal distribution and variance homogeneity of model residuals.

### Sequencing datasets

Data were analyzed using the phyloseq package (McMurdie and Holmes 2013), and rarefaction plots (Figure S3, Supporting Information) were created using the function phyloseq.extended:: ggrare. Bacterial and fungal data were rarefied to even depth using the phyloseq:: rarefy_even_depth function resulting in 6651 bacterial and 1896 fungal OTUs, respectively (25% of bacterial and 40% of fungal sequences were lost). α-diversity estimates (Shannon index and observed richness) were calculated using the phyloseq:: estimate_richness function and Pielou’s evenness was obtained by dividing the Shannon index by the natural logarithm of the observed richness. Experimental treatment effects on the respective α-diversity metrics were assessed using linear mixed-effect models as described for the other univariate data.

For β-diversity analyses, we filtered the dataset to remove OTUs with fewer than 10 reads in less than 5% of the samples to reduce the extreme sparsity of microbiome data (Cao et al. 2021). After filtering, the dataset contained 611 730 bacterial and 452 784 fungal sequences clustering into 4138 bacterial and 1200 fungal OTUs. OTU tables were transferred into relative abundances by total sum scaling. Permutational multivariate analysis of variance (PERMANOVA) (Anderson 2001) using the vegan:: adonis2 function (Oksanen et al. 2022) based on Bray–Curtis distances was used to test treatment effects (FS, FI, and their interaction) at 9999 permutations restricted on the field blocks. The homogeneity of multivariate dispersion in the treatment groups was confirmed using PERMDISP (Anderson et al. 2006) implemented through the betadisper function in vegan (Oksanen et al. 2022). Pairwise differences between FI within FSs were obtained using the rvaidemoire:: pairwise.perm.manova function (Hervé 2022). To graphically present bacterial and fungal community structures, nonmetric multidimensional scaling (NMDS) was employed (Bray–Curtis distances, k = 3 and trymax = 100) using vegan:: metaMDS (Oksanen et al. 2022). A canonical analysis of principal coordinates (Anderson and Willis 2003) was performed using the CAPdiscrim function implemented in the BiodiversityR package (Kindt and Coe 2005) constraining the function by FS and FI. Joint biplots with the environmental variables correlating with the projections of the ordination were calculated using vegan:: envfit (Oksanen et al. 2022). *P*-values of correlations were corrected for multiple testing based on Benjamini–Hochberg corrections using stats:: p.adjust (R Core Team 2021). The graphical presentation was restricted to variables that exceeded an *a priori* threshold of r^2^ > 0.6 and adjusted *P*-values < .01.

Each bacterial OTU was classified into oligotroph or copitroph lifestyle based on its ribosomal RNA (*rrn)* copy number (oligotrophy <0.5, copiotroph ≥5 according to Bledsoe et al. 2020) using the RDP classifier (Wang et al. 2007) and the NCBI taxonomy integrated within the *rrn* operon database (Stoddard et al. 2015). The classification was performed at the lowest possible taxonomic rank, provided in the *rrn* operon database. The relative abundance of copiotroph, oligotroph, and unclassified OTUs was summed for each soil sample to calculate the copiotroph-to-oligotroph ratio within a soil bacterial community. To retrieve information about fungal trophic modes, the FUNGuild database and its bioinformatics script were used (Nguyen et al. 2016) leading to approximately 50% of OTUs being classified (with confidence levels of “possible,” “probable,” and “highly probable”). The relative abundance of OTUs assigned to a specific trophic mode or a combination of trophic modes was summed per sample. Experimental treatment effects on the copiotroph-to-oligotroph ratio as well as on the relative abundance of fungal trophic modes were assessed with linear mixed-effect models as described for the univariate data.

We finally screened the data for OTUs specifically associated with the different FSs and FI. For this test, we removed sequences with fewer than 20 reads in less than 10% of the samples for bacteria and fewer than 50 reads in less than 25% of the samples for fungi. The indicspecies:: multipatt function (De Cáceres and Legendre 2009) was employed to identify OTUs associated with one or more treatment combinations (10^4^ permutations, “r.g” function to correct for unequal group sizes) (Dufrêne and Legendre 1997, De Cáceres et al. 2012). Multiple testing correction was performed by calculating *q*-values using the qvalue package (Storey et al. 2021). A bi-partite association network between treatment groups and statistically associated bacterial and fungal indicator OTUs was generated using Cytoscape v3.9.1 (Shannon et al. 2003) with treatments as source nodes, OTUs as target nodes, and association strength as connecting edges. Only treatment-OTU associations with *q* < 0.05 were selected and the network was built using the Allegro Fruchterman–Reingold layout with edges weighted by association strength (normalized square-root transformed).

## Results

I. Geochemical and biological soil quality indicators

**Figure 1.**
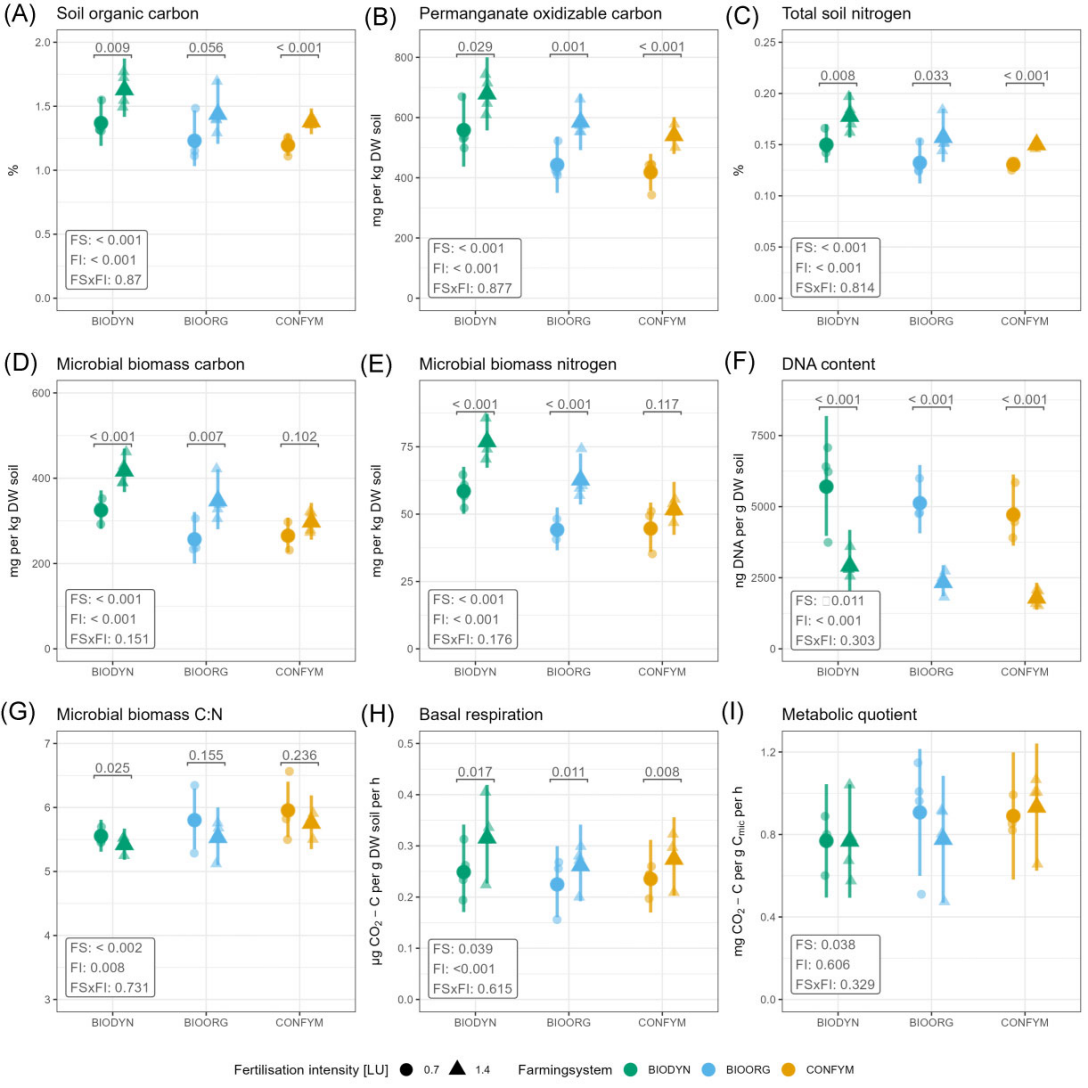
Treatment effects on chemical and biological soil quality indicators. Plots show raw data (semitransparent dots) and estimated marginal means with 95% confidence intervals of linear mixed-effect models assessing treatment effects on soil quality indicators. *P*-values for FI and FS and their interaction are presented in boxes. Statistical significance of planned contrasts between FI within FSs is indicated on top of each comparison if significant FI, or FI × FS main effects were present. Shapes represent FI: 0.7 = 0.7 LU, 1.4 = 1.4 LU. Colors represent the FSs: BIODYN = biodynamic, BIOORG = bioorganic, CONFYM = conventional.

C_org_, PoxC, and N_tot_ were elevated under 1.4 LU compared to 0.7 LU (Fig. 1A–C). Planned contrasts between FI within FSs revealed significantly higher values for C_org_, PoxC, and N_tot_, in high- compared to low-input plots except for C_org_ in BIOORG. FSs differed in C_org_, N_tot_, and PoxC contents with the highest values in BIODYN, followed by BIOORG and CONFYM (Fig. 1A–C; Table S2, Supporting Information). Higher C_mic_ and N_mic_ values were identified under 1.4 LU compared to 0.7 LU (Fig. 1D and E) with planned contrasts revealing significant differences between FI in BIODYN and BIOORG but not in CONFYM. FSs differed in microbial biomasses with the highest values in BIODYN, followed by BIOORG and CONFYM (Fig. 1F; Table S2, Supporting Information). Higher soil DNA content was found in 0.7 LU compared to 1.4 LU plots with planned contrasts for FI being significant in all FSs (Fig. 1F). FSs also differed in their DNA content with the highest values in BIODYN, followed by BIOORG and CONFYM (Fig. 1F; Table S2, Supporting Information). Microbial biomass C:N was slightly, but statistically significant, elevated at 0.7 LU compared to 1.4 LU with planned contrasts revealing significant differences between FI only for BIODYN (Fig. 1G). Moreover, FSs differed in their microbial biomass C:N with the highest values in CONFYM followed by BIOORG and BIODYN (Fig. 1G; Table S2, Supporting Information). Basal respiration was higher at 1.4 LU compared to 0.7 LU and planned contrasts revealed significant differences between FI for all FSs (Fig. 1H). The metabolic quotient remained unaffected by FI but was affected by FSs, with the lowest values in BIODYN (Fig. 1I).

II. Soil bacterial and fungal communities

### Data overview

After removing any nonbacterial sequences and filtering rare OTUs (prevalent in less than 5% of the samples and with fewer than 10 reads), the bacterial dataset contained 4138 OTUs. The five most abundant phyla were *Proteobacteria, Actinobacteria, Verrucomicrobia, Planctomycetes*, and *Acidobacteria* (Figure S4A, Supporting Information). The filtered fungal dataset contained 1200 OTUs and the four most abundant phyla were *Ascomycota, Mortierellomycota, Basidiomycota*, and *Chytridiomycota* (Figure S4B, Supporting Information).

### Microbial α- and β-diversity

**Figure 2.**
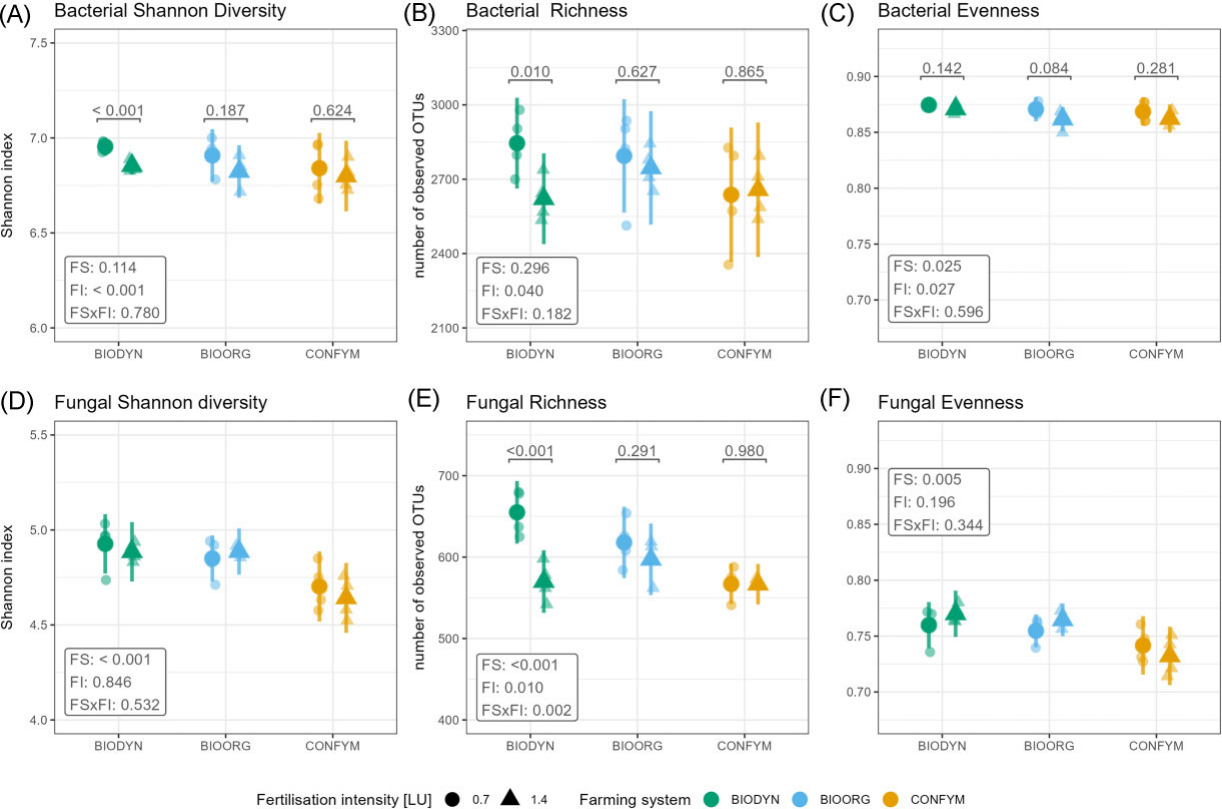
Treatment effects on α-diversity. Plots show raw data (semitransparent dots) and estimated marginal means with 95% confidence intervals of linear mixed-effect models assessing treatment effects on Shannon diversity, observed richness end community evenness. *P*-values for FI and FS and their interaction are presented in boxes. Statistical significance of planned contrasts between FI within FSs is indicated on top of each comparison if significant FI, or FI × FS main effects were found. Shapes represent FI: 0.7 = 0.7 LU, 1.4 = 1.4 LU. Colors represent the FSs: BIODYN = biodynamic, BIOORG = bioorganic, CONFYM = conventional.

For bacteria, ɑ-diversity (Shannon and observed richness) was slightly higher at 0.7 LU compared to 1.4 LU, but differences between FI within FSs were statistically supported only for BIODYN (Fig. 2A and B) with comparable values between FSs (Fig. 2B; Table S2, Supporting Information). Bacterial community evenness was higher in 0.7 LU compared to 1.4 LU, while planned contrasts between FI within FSs revealed no significant differences (Fig. 2B). Bacterial communities were more even under BIODYN compared to BIOORG and CONFYM (Table S2, Supporting Information). Fungal Shannon diversity was comparable in 0.7 LU and 1.4 LU but differed between FSs with higher values for BIODYN and BIOORG compared to CONFYM (Fig. 2D; Table S2, Supporting Information). For fungal richness, an interaction effect between FS and FI was found with significantly increased richness in 0.7 LU compared to 1.4 LU in BIODYN (Fig. 2E) and higher richness in BIOORG and BIODYN compared to CONFYM (Table S2, Supporting Information). Fungal community evenness was unaffected by FI but higher values were observed in BIODYN and BIOORG compared to CONFYM (Fig. 2F; Table S2, Supporting Information).

The PERMANOVA test identified distinct bacterial and fungal community structures in 0.7 LU and 1.4 LU plots and different FSs (Table 2). The FS explained around twice as much data variation (bacteria: 24%, fungi: 27%) compared to FI (bacteria: 12%, fungi: 13%) (Table 2). For bacteria, the pairwise PERMANOVA revealed trends for differences between FI in BIOORG (*P* = .085), and BIODYN (*P* = .085) but not in CONFYM (*P* = .259) whereas, for fungi, communities differed between FI in BIOORG (*P* = .029), BIODYN (*P* = .028), and CONFYM (*P* = .030).

**Table 2.** Treatment effects on β-diversity. Treatment effects were assessed by PERMANOVA (9999 permutations) on a distance matrix (Bray–Curtis) based on filtered and total sum scaled OTU tables.

	Bacteria				Fungi			
	Df	*R* ^2^	F	*P*	Df	*R* ^2^	F	*P*
FS	1	0.24	3.70	0.004	1	0.27	4.71	< 0.001
FI	2	0.12	3.61	< 0.001	2	0.13	4.55	< 0.001
FS × FI	2	0.05	0.75	0.532	2	0.07	1.14	0.2911

**Figure 3.**
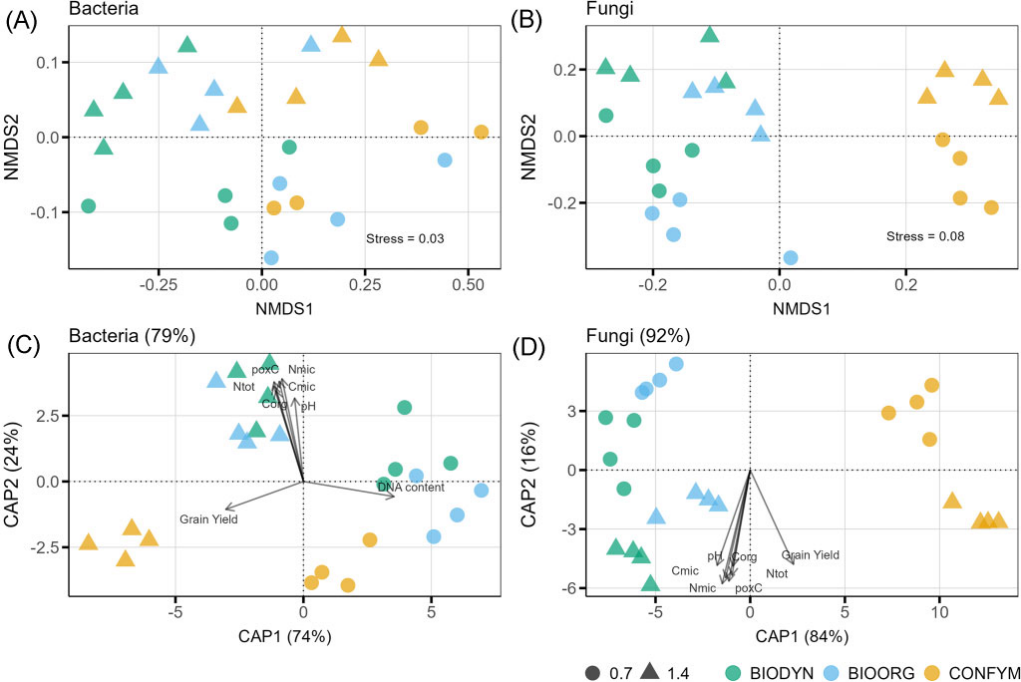
NMDS and canonical analyses of principal coordinates (CAP) of bacterial and fungal communities. NMDS ordinations of bacterial (A) and fungal (B) communities based on total sum scaled OTU tables and Bray–Curtis distances. The corresponding CAPs for bacteria (C) and fungi (D) are constrained by FS and fertilization. The overall reclassification success rate is given in the title for each CAP ordination. Arrows in (C) and (D) represent correlations of soil properties with ordination scores and are scaled according to correlation strength. Only correlations with *r*^2^ ≥ 0.6 and *q*-values < 0.01 are shown (full list in Table S3, Supporting Information). Shapes represent FI: 0.7 = 0.7 LU, 1.4 = 1.4 LU. Colors represent the FSs: BIODYN = biodynamic, BIOORG = bioorganic, CONFYM = conventional.

The NMDS of bacterial and fungal communities confirmed effects identified by the PERMANOVA, with FSs separating along the first axis, and FI separating along the second axis (Fig. 3A and B). Microbial community structure differed between FSs along the gradient of BIODYN-BIOORG-CONFYM, whereas, in the fungal dataset, BIODYN and BIOORG represent one cluster and CONFYM another one (Fig. 3A and B). These patterns were also confirmed by the CAP ordinations with reclassification values of 79% and 92%, respectively (Fig. 3C and D). In the bacterial and fungal dataset C_mic_, N_mic_, C_org_, PoxC, N_tot_, and pH correlated with the projections of communities from BIODYN and BIOORG at 1.4 LU fertilization (Fig. 3C; Table S3, Supporting Information). For bacteria, DNA content correlated with projections of 0.7 LU systems and grain yield with projections of 1.4 LU CONFYM (Fig. 3C and D).

### Microbial trophic lifestyles and indicative OTUs

**Figure 4.**
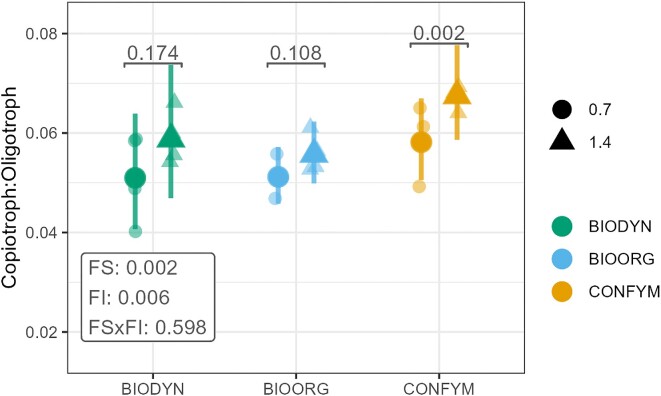
Treatment effects on the bacterial copiotroph-to-oligotroph ratio. The copiotroph-to-oligotroph ratio was calculated based on the cumulative relative abundance of either oligotroph or copiotroph classified bacterial OTUs. The plot shows raw data (semitransparent dots) and estimated marginal means with 95% confidence intervals of linear mixed-effect models assessing treatment effects on the copiotroph-to-oligotroph ratio (based on *rrn* copy numbers). *P*-values for FI and FS and their interaction are presented in boxes. Statistical significance of planned contrasts between FI within FSs is indicated on top of each comparison. Shapes represent FI: 0.7 = 0.7 LU, 1.4 = 1.4 LU. Colors represent the FSs: BIODYN = biodynamic, BIOORG = bioorganic, CONFYM = conventional.

Overall, bacterial communities were oligotroph dominated under all FSs and fertilization levels (Figure S5A, Supporting Information). The copiotroph-to-oligotroph ratio was significantly higher under 1.4 LU compared to 0.7 LU with planned contrasts revealing significant differences between FI only for CONFYM (Fig. 4). BIODYN and BIOORG showed a lower copiotroph-to-oligotroph ratio compared to CONFYM (Table S2, Supporting Information). The distribution of fungal trophic modes showed a more complex picture. The cumulative relative abundance of OTUs associated with a single trophic mode was lowest for the symbiotrophs, followed by pathortophs and saprotrophs. The majority of OTUs, however, were assigned to a combination of several trophic modes or remained unclassified and no clear treatment dependent patterns were observed (Figure S5B, Supporting Information).

**Figure 5.**
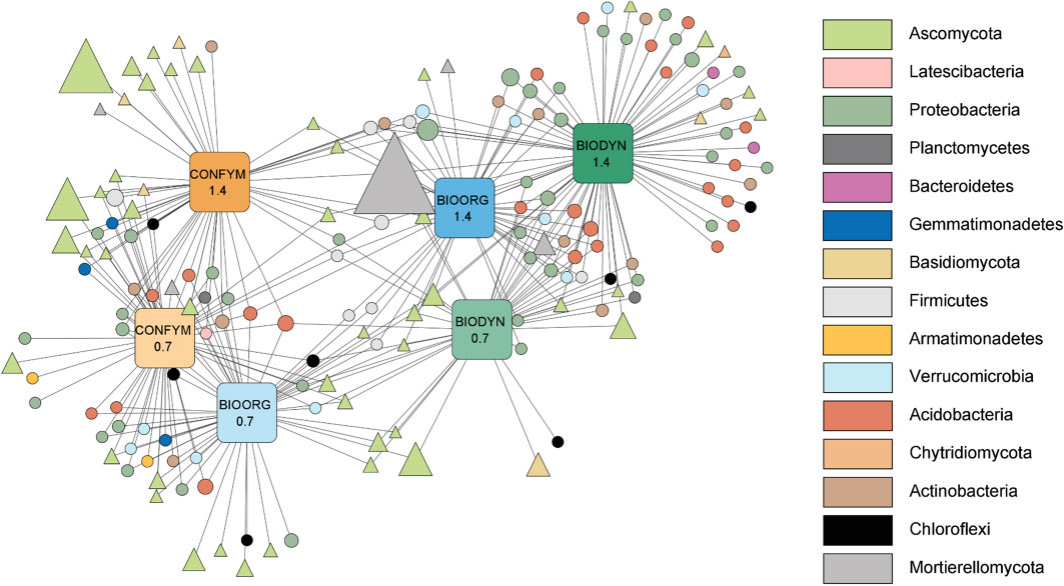
Bi-partite association network of fungal and bacterial indicator OTUs. The network is showing significant (*q* < 0.05) positive associations between treatment groups (FSs × FI) and fungal (diamonds) or bacterial (circles) operational taxonomic units (bOTUs, fOTU) based on indicator species analysis. Node sizes reflect the relative abundance of a given OTUs across all samples in the fungal or bacterial dataset, respectively. The Allegro Fruchterman–Reingold algorithm was applied to construct the network with edges weighted according to the association strength. Nodes outlined in bold represent indicator OTUs specifically associated with high- or low-input and independent of the FS (see Table 3). Node colors represent different phyla. BIODYN = biodynamic (green), BIOORG = bioorganic (blue), and CONFYM = conventional (orange). 0.7 = 0.7 LU, 1.4 = 1.4 LUs. Nodes without annotation on the phylum level were excluded. A full list of indicator species, their taxonomic information, and attributed lifestyles are given in Table S4 (Supporting Information).

A total of 110 bacterial and 67 fungal OTUs (bOTU and fOTU, respectively) were associated (*q* < 0.05) with specific treatments or treatment combinations (Table S4, Supporting Information). The bipartite network constructed based on these indicator OTUs showed 0.7 LU and 1.4 LU to be separated from each other as well as FSs (Fig. 5). FI of each FS grouped closely except for BIOORG, where a bidirectional formation was observed with 0.7 LU BIOORG closely associated with the CONFYM systems and 1.4 LU BIOORG associated with the BIODYN systems (Fig. 5).

When exclusively looking at OTUs indicative of FI and simultaneously associated with all three FSs of the respective FI, four indicator bOTUs and zero fOTU were identified for 1.4 LU and three bOTUs and two fOTUs for 0.7 LU systems (Table 3, Fig. 5). Two out of the four bOTUs indicative of 1.4 LU were attributed to a copiotrophic lifestyle, while none of the bOTUs indicative of 0.7 LU systems was classified as copiotroph (Table 3). The two fOTUs indicative of 0.7 LU could not be assigned to a trophic mode.

**Table 3.** List of indicator OTUs associated with contrasting FI. Given are fungal and bacterial indicator OTUs that are positively associated (*q* < 0.05) with a FI level and also associated with all three FSs of the respective FI. The taxonomic assignment is provided at the lowest possible level and relative abundance across the entire dataset is given. Attributed lifestyles are determined based on *rnn* copy numbers (oligotrophy < 0.5, copiotroph ≥ 5) to the lowest possible taxonomic rank provided in the rnnDB. The fungal lifestyles of the two fOTUs remained unclassified using FUNGuild. Relative abundance is given in %.

Treatment association	OTU	Relative abundance	Kingdom	Phylum	Class	Order	Family	Genus	*q*-value	Associated lifestyle	Mean *rrn* copies	STDEV	Entries in rrnDB
1.4 LU	bOTU17	0.624	Bacteria	*Proteobacteria*	*Alphaproteobacteria*	*Rhizobiales*	*Rhodobiaceae*		0.010	Oligotroph	1	0	2
	bOTU23	0.150	Bacteria	*Firmicutes*	*Bacilli*	*Bacillales*	*Bacillaceae*	*Bacillus*	0.010	Copiotroph	10.31	2.4	798
	bOTU6	0.088	Bacteria	*Firmicutes*	*Bacilli*	*Bacillales*	*Bacillaceae*	*Bacillus*	0.010	Copiotroph	10.31	2.4	798
	bOTU1379	0.036	Bacteria	*Actinobacteria*	*Actinobacteria*	*Micrococcales*	*Promicromonosporaceae*		0.013	Oligotroph	3.09	0.3	11
0.7 LU	bOTU972	0.081	Bacteria	*Chloroflexi*	*Chloroflexia*	*Chloroflexales*	*Roseiflexaceae*	*Roseiflexus*	0.034	Oligotroph	2	0	2
	bOTU473	0.025	Bacteria	*Proteobacteria*	*Deltaproteobacteria*	*Desulfuromodales*	*Geobacteraceae*	*Geobacter*	0.028	Oligotroph	2.64	0.93	14
	bOTU2583	0.006	Bacteria	*Verrucomicrobia*	*OPB35_soil_group*				0.038	Oligotroph	2.49	0.84	41
	fOTU62	0.333	Fungi	*Ascomycota*	*Dothideomycetes*	*Pleosporales*			0.044	Unlassified	–	–	–
	fOTU82	0.216	Fungi	*Ascomycota*	*Sordariomycetes*	*Sordariales*	*Chaetomiaceae*		0.031	Unlassified	–	–	–

The cluster of nodes indicative for the two 1.4 LU organic systems was built exclusively of bacterial OTUs while the cluster of nodes indicative for 0.7 LU organic systems was built of fungal OTUs only (Fig. 5; Table S4, Supporting Information). Overall, fungal indicator OTUs were more often associated with 0.7 LU systems and CONFYM compared to 1.4 LU BIODYN and 1.4 LU BIOORG (Fig. 5; Table S4, Supporting Information).

## Discussion

The need for sustainable agroecosystems combined with the demand for decreasing livestock density (Köninger et al. 2021, Eisen and Brown 2022) calls to further identify how a reduction of animal manure-based FI affects soil microbial communities. By using soil samples obtained from winter wheat plots of a 42-year-old long-term trial, we found that high inputs (1.4 LU) of animal manures in different FSs (i) increased soil C and soil N contents, (ii) tended to decrease soil bacterial alpha-diversity, and (iii) altered soil microbial community structure as compared to low-input systems (0.7 LU) across all FSs.

I. High-input systems increase soil C and N content compared to low-input FSs

Chemical soil properties provide information on the nutritional status of the soil representing the microbial habitat (Voroney 2007). The amount of C introduced via organic fertilization directly, and indirectly via plant roots and rhizodeposition, determines the soil C contents with higher values under high- compared to low-input. This finding is consistent with our expectation, and what has been previously shown at other experimental sites (e.g. Francioli et al. 2016, Ma et al. 2020a). The higher soil C contents in BIODYN may be related to the quality of introduced C as this system receives composted manure with higher recalcitrance compared to BIOORG receiving rotten and CONFYM receiving stacked manure. Given the strong link between soil C and other geochemical parameters, similar patterns were observed for PoxC and N content.

Overall, microbial biomass C and N were elevated under high- compared to low-input systems, which was also observed in another long-term trial (Ma et al. 2020b). This is partially in line with a recent meta-analysis (Ren et al. 2019) showing that overall organic fertilizer increases microbial biomass, while more specific the effect of FYM was highly variable and remained insignificant.

It is known that the majority of soil microbes are heterotrophs that obtain energy and nutrients from organic matter and that their growth and activity depend on the concentration and supply rate of bioavailable organic material (Coleman et al. 2004). We indeed observed increased basal respiration rates in high-input compared to low-input systems. As the metabolic quotient, a measure of microbial substrate use efficiency (Wardle and Ghani 1995), remained unaffected by FI, we assume that the increased soil basal respiration under high-input systems is more strongly driven by the observed increased abundance of microbes than by distinct C-mineralization machinery.

Usually, soil DNA content and microbial biomass are highly correlated (Semenov et al. 2018) but surprisingly, we found higher DNA contents in soil sampled from low- compared to high-input systems. A possible explanation might be the chronic P-depletion of the low-input systems in the DOK trial (Jarosch et al., submitted) likely resulting in freer and more positively charged soil particles acting as putative binding places for passively or actively released extracellular DNA (Nagler et al. 2018).

II. Distinct microbial communities under low- compared to the high-input FS

The importance of soil microbial diversity for maintaining ecosystem functioning (Delgado-Baquerizo et al. 2016) and stability (Wagg et al. 2021) depicts the need to better understand factors shaping microbial communities in agroecosystems to counteract soil degradation (Cavicchioli et al. 2019, Zhou et al. 2020) and to preserve or restore agroecosystem functioning in the future.

Generally, the effect of fertilization type (organic, mineral, or mixed) on soil microbes is complex and contrasting trends were reported in the literature depending on the fertilizer origin and amount, and the edaphic factors. A recent meta-analysis by Shu et al. (2022) synthesized that organic amendments significantly increased microbial diversity and shifted microbial community structure compared to mineral-only fertilization. However, the performance of microbial α-diversity varied substantially with organic amendment types, microbial groups, and changes in soil pH. Exemplary more in detail, Francioli et al. (2016) observed that soils amended with FYM and/or a mixture of FYM and mineral fertilizer are characterized by an increased nutritional status, higher microbial biomass, higher bacterial α-diversity, and distinct microbial community structure as compared to exclusively mineral fertilized soils. Hartmann et al. (2015) showed in the DOK trial, that organically fertilized FSs increase the microbial richness and shift community structure when compared to exclusively mineral fertilized systems.

Based on these observations, we hypothesized to find a higher α-diversity in low- compared to high-input systems because a diminished nutrient availability under low-input likely requires a taxonomically and functionally more versatile community to degrade complex recalcitrant C and retrieve energy from other sources than the organic fertilizer. However, we found bacterial and fungal α-diversity to be only marginally affected by contrasting fertilizer intensity. The small effect of FI on α-diversity is largely in line with a study by Wang et al. (2021) showing bacterial, fungal, and archaeal α-diversity to be unaffected over an organic FI gradient in a grassland soil. Whether the herein observed weak increase in α-diversity under low- compared to high-input BIODYN translates into distinct functioning has not been studied and remains speculative.

As hypothesized, we found distinct fungal and bacterial community structures under high- and low-input in all FSs. The correlations of C_org_, PoxC, and total N content with the projections of the ordination of bacterial and fungal community structures under high-input underpin the possible shift of microbial lifestyles due to contrasting nutrient availabilities (Kim et al. 2021). Characterizing bacterial communities based on their putative ecological lifestyles, we identified oligotroph-dominated communities in all FSs and FI. This may be related to the fact that copiotroph microbes thrive under nutrient-rich conditions; however, we collected the samples before the spring fertilization. We presume that the putative copiotroph-classified OTUs would increase in relative abundance after spring fertilization events. Moreover, the overall low abundance of copiotrophic OTUs might also result from the rather conservative threshold set for *rrn* copy numbers to classify copio- vs. oligotrophic OTUs (Bledsoe et al. 2020).

The ratio of putative copiotroph- to oligotroph-classified bacteria was slightly higher under high- compared to low-input as well as in conventional compared to organic FSs, pointing out a shift towards a more copiotroph community under high-input and CONFYM systems. Fungi cannot be classified into ecological lifestyles based on their *rrn* copy numbers but they usually show more oligotrophic features than bacteria (Ho et al. 2017) and are attributed with a strong capability of degrading recalcitrant polymers to gain nutrients and energy (Van der Wal et al. 2013). The distribution of fungal trophic modes based on FUNGuild annotations did not yield clear results as half of the OTUs remained unclassified and many OTUs could not be categorized into one but rather a combination of serval trophic modes. However, the slightly higher C_mic_ to N_mic_ ratio in CONFYM and low-input systems as well as the more frequent association of indicative fungal OTUs with low-input and CONFYM in the bi-partite network support the idea of a fungal dominance in the more oligotrophic habitats.

Looking at indicator OTUs associated with either low- or high-input systems, but compellingly also associated with all three FSs of the respective FI level, we found two fungal indicators with described oligotrophic features and three oligotroph classified bacterial OTUs indicative of low-input systems. In contrast, we found no fungal and four bacterial OTUs to be indicative of high-input systems of which two were classified as putative copiotrophs. The copiotrophic indicators associated with high-input systems are both assigned to the endospore-forming Gram-positive bacterial genus *Bacillus* (Nicholson et al. 2000, Nicholson 2002), of which many members are known for their plant growth promoting features (Goswami et al. 2016, Saxena et al. 2020).

The bacterial indicator OTUs associated with low-input systems were all classified as oligotrophs and were attributed to diverse ways of energy metabolism. For example, bOTU473 is assigned to *Geobacter*, which is known for establishing electrical contacts with extracellular electron acceptors and other organisms, and acts as the primary agent for coupling the oxidation of organic compounds to the reduction of insoluble Fe(III) and Mn(IV) oxides in many soils and sediments (Lovley et al. 2011, Yi et al. 2013, Holmes et al. 2017). bOTU972 was assigned to *Roseiflexus*, which can grow photoheterotrophically under light and chemoheterotrophically in the dark (Hanada 2014).

In summary, indicator OTUs associated with high-input systems showed more copiotrophic features than indicators associated with low-input systems, which is in line with the enhanced copiotroph-to-oligotroph ratio under high-input systems. The higher availability of nutrients and the more easily available C in high-input systems may have depleted some oligotrophic functional members and enriched a few copiotrophic members potentially leading to a higher nutrient dependency. We found indications that the diminished nutrient availability in the low-input systems selects for a functionally versatile microbial community being able to degrade complex recalcitrant C and retrieve energy and nutrients from other sources under nutritional constraints.

Nonetheless, we can only speculate about the possible ecological functions of the identified indicator OTUs, and the classification of bacterial lifestyles is an approximation only. Extended analyses such as the profiling of microbial metabolic capacity (Creamer et al. 2016), proteomics (Qian and Hettich 2017), shotgun metagenomics (Vogel et al. 2009), and combinations thereof (Martinez-Alonso et al. 2019) might further reveal distinct effects of FI on soil multifunctionality influencing agronomic variables.

## Conclusion

We showed that a reduction of FYM application from 1.4 LU to 0.7 LU translates into lower soil C and N content selecting for distinct microbial communities in bioorganic, biodynamic, and conventionally farmed winter wheat plots. Thus, a decrease in livestock density and herewith-associated reduced levels of FYM inputs in organic and integrated FSs change the habitat quality of agricultural soils and affect soil microbial communities. The reduced nutrient availability under low-input selects for more oligotrophic microbiomes likely more efficiently obtaining nutrients from various carbon sources; a potentially beneficial trait considering future agroecosystems.

## Supplementary Material

fiad046_Supplemental_Files
