# Supplementary material for: Soil microbial communities are sensitive to differences in fertilization intensity in organic and conventional farming systems
Source: FEMS Microbiol Ecol. 2023 May 9;99(6):fiad046. doi: 10.1093/femsec/fiad046 (PMC10236208; doi:10.1093/femsec/fiad046)
Supplement: fiad046_Supplemental_Files [file fiad046_supplemental_files.zip › Supplementary_Figure_legends.docx]

**Supplementary Figure 1. Field setup of the DOK trial modified from Krause et al., 2022.** Each year three crops are cultivated in iterations labelled as A, B, and C. N= NOFERT (unfertilized control), D= BIODYN (biodynamic); O= BIOORG (bioorganic); K= CONFYM (conventional with farmyard manure); M= CONMIN (conventional mineral fertilized). Fertilization intensities: 0.7 and 1.4 correspond to organic fertilization at 0.7 and 1.4 livestock units per hectare.

**Supplementary Figure 2. Composition of bacterial and fungal mock communities.** Relative abundance of sequenced bacterial and fungal mock communities (left) versus the expected abundance (right).

**Supplementary Figure 3. Rarefaction plots of the bacterial and fungal sequencing dataset before subsampling and filtering.** Samples are colored according to farming systems: BIODYN= biodynamic, BIOORG= bioorganic, CONFYM= conventional. Vertical lines indicate the sample with the lowest sequence counts.

**Supplementary Figure 4. Mean relative abundance of operational taxonomic units aggregated on the phylum level for bacteria (A) and fungi (B).** BIODYN= biodynamic, BIOORG= bioorganic, CONFYM= conventional. Fertilization intensities are given in livestock units (LU).

**Supplementary Figure 5. Distributions of putative trophic modes in bacterial fungal communities.** A: Cumulative relative abundances of putative oligotroph, copiotroph, and unclassified bacterial OTUs with lifestyles determined based on *rnn* copy numbers (oligotrophy <0.5, copiotroph ≥5) (A). B: Cumulative relative abundances of distinct putative fungal trophic modes classified using FunGuild
