# Supplementary material for: Soil microbial communities are sensitive to differences in fertilization intensity in organic and conventional farming systems
Source: FEMS Microbiol Ecol. 2023 May 9;99(6):fiad046. doi: 10.1093/femsec/fiad046 (PMC10236208; doi:10.1093/femsec/fiad046)
Supplement: fiad046_Supplemental_Files [file fiad046_supplemental_files.zip › Supplementary_Table1.docx]

**Supplementary Table 1. Primer sequences and cycling conditions used for amplicon production for bacterial 16S rRNA (V3-V4 region) and fungal (ITS2 region) community characterization**.

| **Target** | **Name** | **Sequence** | **Final concentration** | **Cycling conditions** | **Reference** |
| --- | --- | --- | --- | --- | --- |
| V3-V4 | 341F | CCTAYGGGDBGCWSCAG | 0.2 mM | Initial denaturation: 95 ° C, 3 ‘  denaturation: 95° C, 20’’  Annealing: 58 ° C , C 20’’ x 30  Extension: 72 ° C, 40 ‘’  Final extension: 72 ° C, 10 ‘ | Frey et al. 2016 |
|  | 806R | GGACTACNVGGGTHTCTAAT | 0.2 mM |  |  |
| ITS2 | ITS3ngsmix1-5 | CAN CGA TGA AGA ACG YRG | 0.2 mM | Initial denaturation: 95 ° C, 3 ‘  denaturation: 95° C, 20’’  Annealing: 58 ° C , C 20’’ x 34  Extension: 72 ° C, 40 ‘’  Final extension: 72 ° C, 10 ‘ | Thedersoo and Lindahl 2016 |
|  | ITS4ngsUni | CCT SCS CTT ANT DAT ATG C | 0.2 mM |  |  |
