# Supplementary material for: Soil microbial communities are sensitive to differences in fertilization intensity in organic and conventional farming systems
Source: FEMS Microbiol Ecol. 2023 May 9;99(6):fiad046. doi: 10.1093/femsec/fiad046 (PMC10236208; doi:10.1093/femsec/fiad046)
Supplement: fiad046_Supplemental_Files [file fiad046_supplemental_files.zip › Supplementary_Table2.docx]

**Supplementary Table 2. Estimated means of farming systems and significance planned contrast between farming systems based on linear mixed effect models assessing treatment effects.**

|  | estimated means | | | significance of planned contrast | | | |
| --- | --- | --- | --- | --- | --- | --- | --- |
|  | BIODYN | BIOORG | CONFYM | BIDYN *vs* BIOORG | | BIODYN *vs* CONFYM | BIOORG *vs* CONFYM |
| Soil organic C | 1.49 | 1.33 | 1.28 | 0.041 | < 0.001 | | 0.384 |
| PoxC | 619 | 514 | 479 | 0.006 | < 0.001 | | 0.103 |
| Total soil N | 0.163 | 0.144 | 0.14 | 0.023 | < 0.001 | | 0.442 |
| Microbial biomass C | 369 | 300 | 281 | 0.002 | < 0.001 | | 0.278 |
| Microbial biomass N | 67.4 | 53 | 48.1 | < 0.001 | < 0.001 | | 0.106 |
| DNA content | 4076 | 3457 | 2903 | 0.105 | 0.011 | | 0.061 |
| Microbial biomass C:N | 5.49 | 5.66 | 5.85 | 0.111 | 0.001 | | 0.139 |
| Basal respiration | 0.53 | 0.493 | 0.504 | 0.038 | 0.105 | | 0.21 |
| Metabolic quotient | 0.769 | 0.842 | 0.911 | 0.255 | 0.041 | | 0.261 |
| Bacterial observed richness | 2733 | 2770 | 2647 | 0.564 | 0.357 | | 0.357 |
| Bacterial Shannon | 6.91 | 6.87 | 6.82 | 0.376 | 0.196 | | 0.386 |
| Bacterial Evennness | 0.872 | 0.866 | 0.866 | 0.046 | 0.046 | | 0.830 |
| Fungal observed richness | 612 | 608 | 567 | 0.703 | < 0.001 | | 0.002 |
| Fungal Shannon | 4.91 | 4.87 | 4.67 | 0.403 | < 0.001 | | < 0.001 |
| Fungal Evenness | 0.77 | 0.76 | 0.74 | 0.367 | 0.006 | | 0.006 |
| Copiotroph:Oligotroph | 0.055 | 0.054 | 0.063 | 0.613 | 0.047 | | 0.002 |
