# Supplementary material for: Soil microbial communities are sensitive to differences in fertilization intensity in organic and conventional farming systems
Source: FEMS Microbiol Ecol. 2023 May 9;99(6):fiad046. doi: 10.1093/femsec/fiad046 (PMC10236208; doi:10.1093/femsec/fiad046)
Supplement: fiad046_Supplemental_Files [file fiad046_supplemental_files.zip › Supplementary_Table3.docx]

**Supplementary Table 3: List of variables correlating with the projections of the CAP ordination (Figure 3).**

|  | **R^2^** | **Adjusted p-value** |
| --- | --- | --- |
| ***Bacteria*** | | |
| Nmic | 0.797 | 0.001 |
| PoxC | 0.790 | 0.001 |
| Cmic | 0.783 | 0.001 |
| Ntot | 0.760 | 0.001 |
| Corg | 0.722 | 0.001 |
| DNA content | 0.716 | 0.001 |
| Grain Yield | 0.642 | 0.001 |
| pH | 0.636 | 0.002 |
| Nmin | 0.384 | 0.005 |
| Cmic:Nmic | 0.247 | 0.018 |
| qCO2 | 0.228 | 0.005 |
| Basal respiration | 0.131 | 0.087 |
| ***Fungi*** | | |
| Nmic | 0.850 | 0.001 |
| poxC | 0.821 | 0.001 |
| Cmic | 0.803 | 0.001 |
| Ntot | 0.775 | 0.001 |
| Grain yield | 0.757 | 0.001 |
| pH | 0.734 | 0.001 |
| Corg | 0.709 | 0.001 |
| DNA content | 0.564 | 0.003 |
| Nmin | 0.399 | 0.002 |
| Cmic:Nmic | 0.308 | 0.002 |
| Basal respiration | 0.301 | 0.001 |
| qCO2 | 0.128 | 0.040 |
| Nmic | 0.850 | 0.001 |
