# Supplementary material for: Soil microbial communities are sensitive to differences in fertilization intensity in organic and conventional farming systems
Source: FEMS Microbiol Ecol. 2023 May 9;99(6):fiad046. doi: 10.1093/femsec/fiad046 (PMC10236208; doi:10.1093/femsec/fiad046)
Supplement: fiad046_Supplemental_Files [file fiad046_supplemental_files.zip › Supplementary_Table4.docx]

**Supplementary Table 4. List of all indicator OTUs. Bacterial (A) and fungal (b) indicator OTUs being positively associated (q< 0.05) with a treatment or treatment group combinations**. The taxonomic assignment is given to the lowest possible level. Attributed bacterial lifestyles are determined based on rnn copy numbers (oligotrophy< 0.5, copiotroph≥ 5) to the lowest possible taxonomic rank the rnnDB had entries. Mean rrn copies, standard deviation (STDEV), and the number of entries in the rrnDB are given as well. Fungal trophic modes were assigned by FUNGuild and confidence ranking is provided.

| **A** | **OTU** | **relative** | **Kingdom** | **Phylum** | **Class** | **Order** | **Family** | **Genus** | **Species** | **q.value** | **Association** | **Lifestyle** | **Mean** | **STDEV** | **Entries** |
| --- | --- | --- | --- | --- | --- | --- | --- | --- | --- | --- | --- | --- | --- | --- | --- |
|  | OTU101 | 0.199 | Bacteria | Proteobacteria | Betaproteobacteria | Burkholderiales | Alcaligenaceae | NA | NA | 0.022 | BIODYN_0.7,BIODYN_1.4,BIOORG_1.4 | Oligotroph | 3.02 | 0.19 | 829 |
|  | OTU1037 | 0.012 | Bacteria | Verrucomicrobia | OPB35_soil_group | NA | NA | NA | NA | 0.049 | BIOORG_0.7,CONFYM_0.7 | Oligotroph | 2.49 | 0.84 | 41 |
|  | OTU106 | 0.141 | Bacteria | Actinobacteria | Acidimicrobiia | Acidimicrobiales | NA | NA | NA | 0.017 | BIOORG_0.7,CONFYM_0.7,CONFYM_1.4 | Oligotroph | 1.67 | 0.58 | 3 |
|  | OTU1062 | 0.018 | Bacteria | Verrucomicrobia | OPB35_soil_group | NA | NA | NA | NA | 0.034 | BIOORG_0.7,CONFYM_0.7 | Oligotroph | 2.49 | 0.84 | 41 |
|  | OTU1084 | 0.007 | Bacteria | Chloroflexi | Chloroflexia | Chloroflexales | FFCH7168 | NA | NA | 0.042 | BIODYN_0.7 | Oligotroph | 2.6 | 0.55 | 5 |
|  | OTU1087 | 0.005 | Bacteria | Chloroflexi | TK10 | NA | NA | NA | NA | 0.042 | CONFYM_0.7,CONFYM_1.4 | Oligotroph | 1.37 | 0.72 | 43 |
|  | OTU1090 | 0.012 | Bacteria | Firmicutes | Bacilli | Bacillales | Thermoactinomycetaceae | Planifilum | NA | 0.041 | BIOORG_0.7,BIOORG_1.4 | Copiotroph | 6.29 | 2.87 | 7 |
|  | OTU1109 | 0.026 | Bacteria | Firmicutes | Bacilli | Bacillales | Thermoactinomycetaceae | Thermoflavimicrobium | NA | 0.028 | BIODYN_0.7,BIODYN_1.4,BIOORG_1.4 | Copiotroph | 6.29 | 2.87 | 7 |
|  | OTU1111 | 0.011 | Bacteria | Proteobacteria | Gammaproteobacteria | Xanthomonadales | Xanthomonadaceae | Thermomonas | NA | 0.035 | BIODYN_1.4 | Oligotroph | 2 | 0 | 5 |
|  | OTU1120 | 0.022 | Bacteria | Gemmatimonadetes | Gemmatimonadetes | Gemmatimonadales | Gemmatimonadaceae | NA | NA | 0.024 | CONFYM_0.7,CONFYM_1.4 | Oligotroph | 1.75 | 0.5 | 4 |
|  | OTU1183 | 0.004 | Bacteria | Proteobacteria | Alphaproteobacteria | Rhodospirillales | I-10 | NA | NA | 0.040 | CONFYM_0.7 | Oligotroph | 4.42 | 2.14 | 131 |
|  | OTU1187 | 0.007 | Bacteria | Chloroflexi | Anaerolineae | Anaerolineales | Anaerolineaceae | NA | NA | 0.022 | BIODYN_0.7,BIODYN_1.4 | Oligotroph | 1.5 | 0.58 | 4 |
|  | OTU1217 | 0.010 | Bacteria | Bacteroidetes | Sphingobacteriia | Sphingobacteriales | Chitinophagaceae | Terrimonas | NA | 0.036 | BIODYN_1.4 | Oligotroph | 2.96 | 1.7 | 25 |
|  | OTU1222 | 0.011 | Bacteria | Firmicutes | Clostridia | Clostridiales | Heliobacteriaceae | Hydrogenispora | NA | 0.010 | BIOORG_0.7,BIOORG_1.4 | Copiotroph | 6 | 5.66 | 2 |
|  | OTU1237 | 0.015 | Bacteria | Firmicutes | Clostridia | Clostridiales | Ruminococcaceae | Ruminococcaceae_UCG-012 | NA | 0.019 | BIOORG_0.7,BIOORG_1.4 | Oligotroph | 4.05 | 1.47 | 19 |
|  | OTU1250 | 0.012 | Bacteria | Acidobacteria | Subgroup_13 | NA | NA | NA | NA | 0.034 | BIOORG_0.7,CONFYM_0.7 | Oligotroph | 1.27 | 0.45 | 26 |
|  | OTU1311 | 0.014 | Bacteria | Proteobacteria | Deltaproteobacteria | NB1-j | NA | NA | NA | 0.046 | BIODYN_1.4 | Oligotroph | 3 | 1.26 | 118 |
|  | OTU1317 | 0.146 | Bacteria | Verrucomicrobia | Spartobacteria | Chthoniobacterales | DA101_soil_group | NA | NA | 0.034 | BIODYN_1.4,CONFYM_1.4 | Oligotroph | 1 | NA | 1 |
|  | OTU1373 | 0.020 | Bacteria | Verrucomicrobia | Opitutae | Opitutales | Opitutaceae | Opitutus | NA | 0.042 | BIODYN_1.4 | Oligotroph | 1 | NA | 1 |
|  | OTU1379 | 0.036 | Bacteria | Actinobacteria | Actinobacteria | Micrococcales | Promicromonosporaceae | NA | NA | 0.013 | BIODYN_1.4,BIOORG_1.4,CONFYM_1.4 | Oligotroph | 3.09 | 0.3 | 11 |
|  | OTU1416 | 0.013 | Bacteria | Verrucomicrobia | OPB35_soil_group | NA | NA | NA | NA | 0.042 | BIODYN_0.7,BIODYN_1.4,BIOORG_1.4 | Oligotroph | 2.49 | 0.84 | 41 |
|  | OTU149 | 0.035 | Bacteria | Proteobacteria | Alphaproteobacteria | Rhodospirillales | KCM-B-15 | NA | NA | 0.028 | CONFYM_0.7 | Oligotroph | 4.42 | 2.14 | 131 |
|  | OTU150 | 0.107 | Bacteria | Proteobacteria | Alphaproteobacteria | Rhizobiales | Hyphomicrobiaceae | Devosia | NA | 0.043 | BIOORG_0.7,CONFYM_0.7,CONFYM_1.4 | Oligotroph | 2.71 | 1.98 | 7 |
|  | OTU1534 | 0.009 | Bacteria | Armatimonadetes | Fimbriimonadia | Fimbriimonadales | Fimbriimonadaceae | NA | NA | 0.034 | CONFYM_0.7 | Oligotroph | 2 | NA | 1 |
|  | OTU1542 | 0.021 | Bacteria | Proteobacteria | Alphaproteobacteria | Rhizobiales | Hyphomicrobiaceae | NA | NA | 0.011 | BIODYN_1.4,BIOORG_1.4 | Oligotroph | 2.09 | 1.31 | 23 |
|  | OTU1558 | 0.018 | Bacteria | Acidobacteria | Holophagae | ABS-19 | NA | NA | NA | 0.041 | BIODYN_1.4 | Oligotroph | 1.27 | 0.45 | 26 |
|  | OTU1561 | 0.004 | Bacteria | Chloroflexi | Ktedonobacteria | C0119 | NA | NA | NA | 0.028 | BIOORG_0.7 | Oligotroph | 4 | NA | 1 |
|  | OTU1564 | 0.014 | Bacteria | Proteobacteria | Deltaproteobacteria | Myxococcales | BIrii41 | NA | NA | 0.028 | BIODYN_1.4 | Oligotroph | 3.36 | 0.82 | 33 |
|  | OTU162 | 0.138 | Bacteria | Acidobacteria | Subgroup | NA | NA | NA | NA | 0.038 | BIOORG_0.7,BIOORG_1.4,CONFYM_0.7,CONFYM_1.4 | Oligotroph | 1.27 | 0.45 | 26 |
|  | OTU165 | 0.053 | Bacteria | Proteobacteria | Betaproteobacteria | NA | NA | NA | NA | 0.046 | BIOORG_0.7,CONFYM_0.7,CONFYM_1.4 | Oligotroph | 3.64 | 1.26 | 1847 |
|  | OTU1676 | 0.017 | Bacteria | Actinobacteria | Actinobacteria | Kineosporiales | NA | NA | NA | 0.021 | BIOORG_0.7,CONFYM_0.7 | Oligotroph | 4 | NA | 1 |
|  | OTU17 | 0.624 | Bacteria | Proteobacteria | Alphaproteobacteria | Rhizobiales | Rhodobiaceae | NA | NA | 0.010 | BIODYN_1.4,BIOORG_1.4,CONFYM_1.4 | Oligotroph | 1 | 0 | 2 |
|  | OTU173 | 0.104 | Bacteria | Proteobacteria | Alphaproteobacteria | Sphingomonadales | Erythrobacteraceae | NA | NA | 0.010 | CONFYM_0.7,CONFYM_1.4 | Oligotroph | 1.33 | 0.48 | 30 |
|  | OTU178 | 0.140 | Bacteria | Acidobacteria | Subgroup | NA | NA | NA | NA | 0.022 | BIODYN_0.7,BIODYN_1.4,BIOORG_1.4 | Oligotroph | 1.27 | 0.45 | 26 |
|  | OTU1849 | 0.016 | Bacteria | Acidobacteria | Subgroup | NA | NA | NA | NA | 0.042 | BIODYN_1.4 | Oligotroph | 1.27 | 0.45 | 26 |
|  | OTU186 | 0.145 | Bacteria | Proteobacteria | Alphaproteobacteria | Rhizobiales | Hyphomicrobiaceae | Pedomicrobium | NA | 0.026 | BIODYN_1.4,BIOORG_1.4 | Oligotroph | 2.09 | 1.31 | 23 |
|  | OTU1915 | 0.052 | Bacteria | Acidobacteria | Holophagae | Subgroup | NA | NA | NA | 0.022 | BIODYN_0.7,BIODYN_1.4,BIOORG_1.4 | Oligotroph | 1.27 | 0.45 | 26 |
|  | OTU194 | 0.074 | Bacteria | Acidobacteria | Solibacteres | Solibacterales | Bryobacter | NA | NA | 0.046 | BIOORG_0.7,CONFYM_0.7,CONFYM_1.4 | Oligotroph | 1.27 | 0.45 | 26 |
|  | OTU198 | 0.249 | Bacteria | Acidobacteria | Subgroup | NA | NA | NA | NA | 0.022 | BIOORG_0.7,CONFYM_0.7 | Oligotroph | 1.27 | 0.45 | 26 |
|  | OTU2003 | 0.004 | Bacteria | Proteobacteria | Deltaproteobacteria | Myxococcales | Haliangiaceae | Haliangium | NA | 0.042 | BIODYN_1.4 | Oligotroph | 2 | NA | 1 |
|  | OTU2063 | 0.007 | Bacteria | Proteobacteria | Alphaproteobacteria | Rhizobiales | Hyphomicrobiaceae | Rhodomicrobium | NA | 0.042 | BIODYN_1.4 | Oligotroph | 2 | NA | 1 |
|  | OTU2089 | 0.011 | Bacteria | Acidobacteria | Subgroup | NA | NA | NA | NA | 0.041 | BIODYN_1.4 | Oligotroph | 1.27 | 0.45 | 26 |
|  | OTU209 | 0.031 | Bacteria | Gemmatimonadetes | Gemmatimonadetes | Gemmatimonadales | Gemmatimonadaceae | NA | NA | 0.040 | BIOORG_0.7,CONFYM_0.7 | Oligotroph | 1.75 | 0.5 | 4 |
|  | OTU2105 | 0.017 | Bacteria | Planctomycetes | OM190 | NA | NA | NA | NA | 0.039 | BIODYN_0.7,BIODYN_1.4 | Oligotroph | 2.19 | 1.35 | 47 |
|  | OTU2164 | 0.006 | Bacteria | Acidobacteria | Subgroup_25 | NA | NA | NA | NA | 0.042 | BIODYN_1.4 | Oligotroph | 1.27 | 0.45 | 26 |
|  | OTU224 | 0.043 | Bacteria | Actinobacteria | Actinobacteria | Frankiales | Sporichthyaceae | NA | NA | 0.046 | BIOORG_0.7,CONFYM_0.7,CONFYM_1.4 | Oligotroph | 1 | NA | 1 |
|  | OTU226 | 0.052 | Bacteria | Acidobacteria | Blastocatellia | Blastocatellales | RB41 | NA | NA | 0.042 | BIODYN_0.7,BIODYN_1.4,BIOORG_1.4 | Oligotroph | 1 | NA | 1 |
|  | OTU2275 | 0.004 | Bacteria | Actinobacteria | Acidimicrobiia | Acidimicrobiales | Iamiaceae | NA | NA | 0.034 | BIODYN_1.4 | Oligotroph | 1 | NA | 1 |
|  | OTU23 | 0.150 | Bacteria | Firmicutes | Bacilli | Bacillales | Bacillaceae | Bacillus | NA | 0.010 | BIODYN_1.4,BIOORG_1.4,CONFYM_1.4 | Copiotroph | 10.31 | 2.4 | 798 |
|  | OTU2393 | 0.013 | Bacteria | Actinobacteria | Actinobacteria | Streptosporangiales | Streptosporangiaceae | Thermopolyspora | NA | 0.041 | BIODYN_1.4,BIOORG_1.4 | Copiotroph | 5.33 | 0.58 | 3 |
|  | OTU2396 | 0.014 | Bacteria | Proteobacteria | Deltaproteobacteria | Myxococcales | Haliangiaceae | Haliangium | NA | 0.050 | BIODYN_0.7,BIOORG_1.4 | Oligotroph | 2 | NA | 1 |
|  | OTU241 | 0.118 | Bacteria | Proteobacteria | Deltaproteobacteria | Desulfurellales | Desulfurellaceae | H16 | NA | 0.030 | BIODYN_0.7,BIODYN_1.4,BIOORG_1.4 | Oligotroph | 2 | 0 | 4 |
|  | OTU2431 | 0.011 | Bacteria | Acidobacteria | Subgroup | NA | NA | NA | NA | 0.028 | BIOORG_0.7,CONFYM_0.7 | Oligotroph | 1.27 | 0.45 | 26 |
|  | OTU2583 | 0.006 | Bacteria | Verrucomicrobia | OPB35_soil_group | NA | NA | NA | NA | 0.038 | BIODYN_0.7,BIOORG_0.7,CONFYM_0.7 | Oligotroph | 2.49 | 0.84 | 41 |
|  | OTU260 | 0.367 | Bacteria | Proteobacteria | Alphaproteobacteria | Sphingomonadales | Sphingomonadaceae | Sphingomonas | NA | 0.023 | BIODYN_1.4,BIOORG_1.4 | Oligotroph | 1.93 | 0.85 | 46 |
|  | OTU2727 | 0.045 | Bacteria | Proteobacteria | Alphaproteobacteria | Sphingomonadales | Sphingomonadaceae | Sphingomonas | NA | 0.028 | BIODYN_0.7,BIODYN_1.4,BIOORG_1.4 | Oligotroph | 1.93 | 0.85 | 46 |
|  | OTU2775 | 0.011 | Bacteria | Latescibacteria | NA | NA | NA | NA | NA | 0.041 | BIOORG_0.7,CONFYM_1.4 | unclassified | NA | NA | NA |
|  | OTU280 | 0.033 | Bacteria | Proteobacteria | Alphaproteobacteria | Rhodospirillales | JG37-AG-20 | NA | NA | 0.024 | CONFYM_0.7,CONFYM_1.4 | Oligotroph | 4.42 | 2.14 | 131 |
|  | OTU2968 | 0.048 | Bacteria | Chloroflexi | KD4-96 | NA | NA | NA | NA | 0.020 | BIOORG_0.7,CONFYM_0.7,CONFYM_1.4 | Oligotroph | 1.37 | 0.72 | 43 |
|  | OTU2992 | 0.033 | Bacteria | Actinobacteria | Actinobacteria | Propionibacteriales | Nocardioidaceae | Nocardioides | NA | 0.041 | BIODYN_1.4 | Oligotroph | 2.59 | 0.97 | 27 |
|  | OTU3030 | 0.004 | Bacteria | Acidobacteria | Holophagae | Subgroup | NA | NA | NA | 0.034 | BIODYN_1.4 | Oligotroph | 1.27 | 0.45 | 26 |
|  | OTU3161 | 0.004 | Bacteria | Proteobacteria | Deltaproteobacteria | Myxococcales | Haliangiaceae | Haliangium | NA | 0.028 | BIODYN_1.4 | Oligotroph | 2 | NA | 1 |
|  | OTU317 | 0.033 | Bacteria | Firmicutes | Erysipelotrichia | Erysipelotrichales | Erysipelotrichaceae | Turicibacter | NA | 0.022 | BIODYN_0.7,BIODYN_1.4,CONFYM_0.7,CONFYM_1.4 | Copiotroph | 10 | 2.83 | 2 |
|  | OTU3265 | 0.066 | Bacteria | Acidobacteria | Subgroup | NA | NA | NA | NA | 0.034 | BIODYN_1.4,BIOORG_1.4 | Oligotroph | 1.27 | 0.45 | 26 |
|  | OTU367 | 0.068 | Bacteria | Actinobacteria | Actinobacteria | Propionibacteriales | Nocardioidaceae | Nocardioides | NA | 0.040 | BIODYN_0.7,BIODYN_1.4 | Oligotroph | 2.59 | 0.97 | 27 |
|  | OTU369 | 0.269 | Bacteria | Acidobacteria | Solibacteres | Solibacterales | Candidatus_Solibacter | NA | NA | 0.043 | BIODYN_0.7,BIOORG_0.7,CONFYM_0.7,CONFYM_1.4 | Oligotroph | 2 | NA | 1 |
|  | OTU3702 | 0.019 | Bacteria | Armatimonadetes | Fimbriimonadia | Fimbriimonadales | Fimbriimonadaceae | NA | NA | 0.034 | BIOORG_0.7,CONFYM_0.7 | Oligotroph | 2 | NA | 1 |
|  | OTU3845 | 0.007 | Bacteria | Actinobacteria | Actinobacteria | NA | NA | NA | NA | 0.043 | BIODYN_1.4,BIOORG_1.4 | Oligotroph | 3.23 | 1.89 | 1975 |
|  | OTU393 | 0.028 | Bacteria | Actinobacteria | Actinobacteria | Micrococcales | Microbacteriaceae | Agromyces | NA | 0.042 | BIODYN_1.4 | Oligotroph | 2.4 | 0.55 | 5 |
|  | OTU40 | 0.201 | Bacteria | Proteobacteria | Alphaproteobacteria | Rhizobiales | Methylobacteriaceae | NA | NA | 0.033 | BIODYN_1.4 | Copiotroph | 5.19 | 2.07 | 31 |
|  | OTU4308 | 0.007 | Bacteria | Proteobacteria | Gammaproteobacteria | Xanthomonadales | Xanthomonadaceae | Arenimonas | NA | 0.038 | BIODYN_0.7,BIOORG_1.4 | Oligotroph | 2.27 | 0.7 | 369 |
|  | OTU44 | 0.212 | Bacteria | Acidobacteria | Subgroup | NA | NA | NA | NA | 0.042 | BIODYN_0.7,BIODYN_1.4,BIOORG_1.4 | Oligotroph | 1.27 | 0.45 | 26 |
|  | OTU453 | 0.019 | Bacteria | Actinobacteria | Actinobacteria | Micrococcales | Micrococcaceae | NA | NA | 0.042 | CONFYM_1.4 | Oligotroph | 4.08 | 1.88 | 91 |
|  | OTU4626 | 0.065 | Bacteria | Acidobacteria | Subgroup | NA | NA | NA | NA | 0.022 | BIOORG_0.7,CONFYM_0.7,CONFYM_1.4 | Oligotroph | 1.27 | 0.45 | 26 |
|  | OTU4639 | 0.026 | Bacteria | Proteobacteria | Betaproteobacteria | Nitrosomonadales | Nitrosomonadaceae | NA | NA | 0.025 | BIODYN_0.7,BIODYN_1.4 | Oligotroph | 1 | 0 | 10 |
|  | OTU471 | 0.166 | Bacteria | Acidobacteria | Subgroup | NA | NA | NA | NA | 0.042 | BIODYN_0.7,BIODYN_1.4,BIOORG_1.4 | Oligotroph | 1.27 | 0.45 | 26 |
|  | OTU473 | 0.025 | Bacteria | Proteobacteria | Deltaproteobacteria | Desulfuromonadales | Geobacteraceae | Geobacter | NA | 0.028 | BIODYN_0.7,BIOORG_0.7,CONFYM_0.7 | Oligotroph | 2.64 | 0.93 | 14 |
|  | OTU4782 | 0.005 | Bacteria | Bacteroidetes | Sphingobacteriia | Sphingobacteriales | Chitinophagaceae | NA | NA | 0.042 | BIODYN_1.4 | Oligotroph | 2.96 | 1.7 | 25 |
|  | OTU48 | 0.352 | Bacteria | Firmicutes | Bacilli | Bacillales | Bacillaceae | Bacillus | NA | 0.010 | CONFYM_0.7,CONFYM_1.4 | Copiotroph | 10.31 | 2.4 | 798 |
|  | OTU5076 | 0.021 | Bacteria | Acidobacteria | Blastocatellia | Blastocatellales | RB41 | NA | NA | 0.028 | BIODYN_1.4 | Oligotroph | 1 | NA | 1 |
|  | OTU5102 | 0.046 | Bacteria | Acidobacteria | Subgroup | NA | NA | NA | NA | 0.043 | BIODYN_1.4 | Oligotroph | 1.27 | 0.45 | 26 |
|  | OTU5180 | 0.014 | Bacteria | Acidobacteria | Subgroup_17 | NA | NA | NA | NA | 0.042 | BIODYN_1.4 | Oligotroph | 1.27 | 0.45 | 26 |
|  | OTU5191 | 0.052 | Bacteria | Proteobacteria | Betaproteobacteria | TRA3-20 | NA | NA | NA | 0.047 | BIODYN_0.7,BIODYN_1.4,BIOORG_1.4 | Oligotroph | 3.64 | 1.26 | 1847 |
|  | OTU52 | 0.185 | Bacteria | Firmicutes | Clostridia | Clostridiales | Peptostreptococcaceae | NA | NA | 0.028 | BIODYN_1.4,CONFYM_0.7,CONFYM_1.4 | Copiotroph | 11.8 | 1.77 | 70 |
|  | OTU5206 | 0.004 | Bacteria | Proteobacteria | Deltaproteobacteria | Myxococcales | Polyangiaceae | NA | NA | 0.028 | BIODYN_1.4 | Oligotroph | 3.83 | 0.41 | 6 |
|  | OTU5230 | 0.005 | Bacteria | Acidobacteria | Subgroup | NA | NA | NA | NA | 0.041 | BIODYN_1.4 | Oligotroph | 1.27 | 0.45 | 26 |
|  | OTU5417 | 0.004 | Bacteria | Chloroflexi | Chloroflexia | Chloroflexales | Roseiflexaceae | Roseiflexus | NA | 0.048 | BIODYN_1.4 | Oligotroph | 2 | 0 | 2 |
|  | OTU5487 | 0.034 | Bacteria | Proteobacteria | Alphaproteobacteria | Sphingomonadales | Erythrobacteraceae | Altererythrobacter | NA | 0.028 | BIODYN_1.4 | Oligotroph | 1.4 | 0.55 | 5 |
|  | OTU552 | 0.025 | Bacteria | Proteobacteria | Alphaproteobacteria | Rhodobacterales | Rhodobacteraceae | NA | NA | 0.028 | BIODYN_0.7,CONFYM_1.4 | Oligotroph | 2.98 | 1.14 | 185 |
|  | OTU5700 | 0.012 | Bacteria | Actinobacteria | Actinobacteria | Propionibacteriales | Nocardioidaceae | Nocardioides | NA | 0.042 | BIODYN_0.7,BIODYN_1.4 | Oligotroph | 2.59 | 0.97 | 27 |
|  | OTU578 | 0.047 | Bacteria | Verrucomicrobia | OPB35_soil_group | NA | NA | NA | NA | 0.046 | BIODYN_1.4 | Oligotroph | 2.49 | 0.84 | 41 |
|  | OTU588 | 0.032 | Bacteria | Proteobacteria | Betaproteobacteria | Nitrosomonadales | Nitrosomonadaceae | NA | NA | 0.022 | BIOORG_0.7,CONFYM_0.7 | Oligotroph | 1 | 0 | 10 |
|  | OTU6 | 0.088 | Bacteria | Firmicutes | Bacilli | Bacillales | Bacillaceae | Bacillus | NA | 0.010 | BIODYN_1.4,BIOORG_1.4,CONFYM_1.4 | Copiotroph | 10.31 | 2.4 | 798 |
|  | OTU6159 | 0.022 | Bacteria | Proteobacteria | Alphaproteobacteria | Rhizobiales | Methylobacteriaceae | Microvirga | Microvirga_aerilata | 0.034 | BIODYN_1.4 | Oligotroph | 3 | 1 | 3 |
|  | OTU62 | 0.150 | Bacteria | Proteobacteria | Betaproteobacteria | TRA3-20 | NA | NA | NA | 0.035 | BIOORG_0.7 | Oligotroph | 3.64 | 1.26 | 1847 |
|  | OTU6564 | 0.016 | Bacteria | Verrucomicrobia | Spartobacteria | Chthoniobacterales | DA101_soil_group | NA | NA | 0.010 | BIODYN_1.4,BIOORG_1.4 | Oligotroph | 1 | NA | 1 |
|  | OTU68 | 0.173 | Bacteria | Proteobacteria | Gammaproteobacteria | Xanthomonadales | NA | NA | NA | 0.032 | BIODYN_1.4,BIOORG_1.4 | Oligotroph | 2.26 | 0.7 | 381 |
|  | OTU6868 | 0.021 | Bacteria | Proteobacteria | Betaproteobacteria | SC-I-84 | NA | NA | NA | 0.038 | BIOORG_0.7,CONFYM_0.7,CONFYM_1.4 | Oligotroph | 3.64 | 1.26 | 1847 |
|  | OTU710 | 0.044 | Bacteria | Gemmatimonadetes | Gemmatimonadetes | Gemmatimonadales | Gemmatimonadaceae | NA | NA | 0.024 | CONFYM_0.7,CONFYM_1.4 | Oligotroph | 1.75 | 0.5 | 4 |
|  | OTU746 | 0.053 | Bacteria | Proteobacteria | Gammaproteobacteria | Xanthomonadales | Acidibacter | NA | NA | 0.046 | BIOORG_0.7,CONFYM_0.7 | Oligotroph | 2.26 | 0.7 | 381 |
|  | OTU803 | 0.015 | Bacteria | Proteobacteria | Alphaproteobacteria | Sphingomonadales | Sphingomonadaceae | Sphingomonas | NA | 0.034 | BIODYN_0.7,BIODYN_1.4,BIOORG_1.4 | Oligotroph | 1.93 | 0.85 | 46 |
|  | OTU834 | 0.011 | Bacteria | Acidobacteria | Blastocatellia | Blastocatellales | Nov 24 | NA | NA | 0.028 | BIODYN_1.4 | Oligotroph | 1 | NA | 1 |
|  | OTU839 | 0.015 | Bacteria | Proteobacteria | Deltaproteobacteria | Desulfurellales | Desulfurellaceae | H16 | NA | 0.034 | BIOORG_0.7,CONFYM_0.7 | Oligotroph | 2 | 0 | 4 |
|  | OTU864 | 0.058 | Bacteria | Proteobacteria | Alphaproteobacteria | Rhizobiales | Xanthobacteraceae | NA | NA | 0.042 | BIOORG_0.7,CONFYM_0.7,CONFYM_1.4 | Oligotroph | 2 | 0.76 | 8 |
|  | OTU886 | 0.026 | Bacteria | Planctomycetes | Phycisphaerae | CPla-3_termite_group | NA | NA | NA | 0.010 | BIOORG_0.7,CONFYM_0.7,CONFYM_1.4 | Oligotroph | 2 | 1 | 5 |
|  | OTU916 | 0.012 | Bacteria | Actinobacteria | Actinobacteria | Micromonosporales | Micromonosporaceae | Rhizocola | NA | 0.040 | BIODYN_0.7,BIODYN_1.4,BIOORG_1.4 | Oligotroph | 3.56 | 1.5 | 27 |
|  | OTU972 | 0.081 | Bacteria | Chloroflexi | Chloroflexia | Chloroflexales | Roseiflexaceae | Roseiflexus | NA | 0.034 | BIODYN_0.7,BIOORG_0.7,CONFYM_0.7 | Oligotroph | 2 | 0 | 2 |
|  | OTU979 | 0.009 | Bacteria | Verrucomicrobia | OPB35_soil_group | NA | NA | NA | NA | 0.022 | BIOORG_0.7,CONFYM_0.7 | Oligotroph | 2.49 | 0.84 | 41 |
|  | OTU986 | 0.066 | Bacteria | Proteobacteria | Gammaproteobacteria | Xanthomonadales | Xanthomonadaceae | Arenimonas | NA | 0.020 | BIOORG_0.7,CONFYM_0.7 | Oligotroph | 2.27 | 0.7 | 369 |
|  | OTU995 | 0.019 | Bacteria | Verrucomicrobia | OPB35_soil_group | NA | NA | NA | NA | 0.034 | BIODYN_0.7,BIODYN_1.4,BIOORG_1.4 | Oligotroph | 2.49 | 0.84 | 41 |
| **B** | OTU | relative | Kingdom | Phylum | Class | Order | Family | Genus | Species | q.value | Association | trophicMode | confidenceRanking | | |
|  | OTU100 | 0.460 | Fungi | Ascomycota | Sordariomycetes | NA | NA | NA | NA | 0.016 | CONFYM_1.4 | unclassified | na |  |  |
|  | OTU105 | 0.340 | Fungi | Ascomycota | NA | NA | NA | NA | NA | 0.016 | CONFYM_1.4 | unclassified | na |  |  |
|  | OTU109 | 0.285 | Fungi | Ascomycota | Sordariomycetes | Microascales | Microascaceae | NA | NA | 0.011 | CONFYM_1.4 | Pathotroph-Saprotroph-Symbiotroph | Possible | |  |
|  | OTU111 | 0.133 | Fungi | NA | NA | NA | NA | NA | NA | 0.002 | BIODYN_0.7,CONFYM_0.7 | unclassified | na |  |  |
|  | OTU1133 | 0.014 | Fungi | NA | NA | NA | NA | NA | NA | 0.026 | CONFYM_0.7,CONFYM_1.4 | unclassified | na |  |  |
|  | OTU130 | 0.212 | Fungi | Ascomycota | Sordariomycetes | Microascales | NA | NA | NA | 0.009 | BIODYN_1.4,CONFYM_0.7,CONFYM_1.4 | unclassified | na |  |  |
|  | OTU1410 | 0.012 | Fungi | Chytridiomycota | Rhizophydiomycetes | Rhizophydiales | NA | NA | NA | 0.012 | BIODYN_1.4 | unclassified | na |  |  |
|  | OTU15 | 1.949 | Fungi | Ascomycota | Sordariomycetes | Sordariales | NA | NA | NA | 0.002 | CONFYM_0.7,CONFYM_1.4 | unclassified | na |  |  |
|  | OTU162 | 0.184 | Fungi | Ascomycota | Sordariomycetes | Coniochaetales | NA | NA | NA | 0.034 | BIODYN_0.7,BIODYN_1.4,BIOORG_1.4 | unclassified | na |  |  |
|  | OTU17 | 1.363 | Fungi | Ascomycota | Sordariomycetes | Sordariales | NA | NA | NA | 0.020 | BIODYN_0.7,BIOORG_0.7 | unclassified | na |  |  |
|  | OTU1705 | 0.051 | Fungi | Ascomycota | Sordariomycetes | Hypocreales | NA | NA | NA | 0.011 | BIOORG_1.4,CONFYM_1.4 | unclassified | na |  |  |
|  | OTU173 | 0.062 | Fungi | Ascomycota | Sordariomycetes | Hypocreales | NA | NA | NA | 0.027 | BIOORG_0.7 | unclassified | na |  |  |
|  | OTU182 | 0.141 | Fungi | Mortierellomycota | Mortierellomycetes | Mortierellales | Mortierellaceae | NA | NA | 0.044 | BIOORG_1.4 | Saprotroph-Symbiotroph | Possible | |  |
|  | OTU183 | 0.202 | Fungi | Mortierellomycota | Mortierellomycetes | Mortierellales | Mortierellaceae | Mortierella | NA | 0.011 | BIOORG_0.7,CONFYM_0.7,CONFYM_1.4 | Saprotroph-Symbiotroph | Probable | |  |
|  | OTU187 | 0.083 | Fungi | NA | NA | NA | NA | NA | NA | 0.011 | CONFYM_1.4 | unclassified | na |  |  |
|  | OTU213 | 0.054 | Fungi | Ascomycota | Dothideomycetes | Pleosporales | NA | NA | NA | 0.005 | BIODYN_0.7,BIOORG_0.7,BIOORG_1.4 | unclassified | na |  |  |
|  | OTU23 | 0.912 | Fungi | Ascomycota | Sordariomycetes | Sordariales | Sordariales_fam_Incertae_sedis | Staphylotrichum | NA | 0.017 | BIODYN_0.7,BIODYN_1.4 | Saprotroph | Probable | |  |
|  | OTU234 | 0.146 | Fungi | Ascomycota | Sordariomycetes | Sordariales | Lasiosphaeriaceae | NA | NA | 0.011 | BIOORG_0.7 | Saprotroph | Probable | |  |
|  | OTU253 | 0.078 | Fungi | Ascomycota | Sordariomycetes | Sordariales | Lasiosphaeriaceae | Cladorrhinum | Cladorrhinum_phialophoroides_SH1505075.08FU | 0.011 | BIODYN_1.4 | Saprotroph | Probable | |  |
|  | OTU26 | 0.587 | Fungi | Ascomycota | Sordariomycetes | Chaetosphaeriales | Chaetosphaeriaceae | NA | NA | 0.032 | CONFYM_0.7 | Saprotroph | Probable | |  |
|  | OTU288 | 0.055 | Fungi | Ascomycota | NA | NA | NA | NA | NA | 0.030 | CONFYM_0.7,CONFYM_1.4 | unclassified | na |  |  |
|  | OTU297 | 0.141 | Fungi | NA | NA | NA | NA | NA | NA | 0.011 | BIODYN_1.4 | unclassified | na |  |  |
|  | OTU33 | 0.654 | Fungi | Ascomycota | NA | NA | NA | NA | NA | 0.011 | BIODYN_0.7,BIODYN_1.4,BIOORG_0.7,BIOORG_1.4 | unclassified | na |  |  |
|  | OTU340 | 0.035 | Fungi | Ascomycota | Sordariomycetes | Sordariales | Chaetomiaceae | Zopfiella | NA | 0.030 | BIODYN_1.4 | Saprotroph | Highly Probable | |  |
|  | OTU346 | 0.108 | Fungi | Ascomycota | Sordariomycetes | Microascales | Microascaceae | NA | NA | 0.004 | BIOORG_1.4,CONFYM_1.4 | Pathotroph-Saprotroph-Symbiotroph | Possible | |  |
|  | OTU354 | 0.034 | Fungi | Ascomycota | Sordariomycetes | Sordariales | NA | NA | NA | 0.011 | BIOORG_1.4 | unclassified | na |  |  |
|  | OTU355 | 0.037 | Fungi | NA | NA | NA | NA | NA | NA | 0.020 | CONFYM_0.7 | unclassified | na |  |  |
|  | OTU36 | 0.980 | Fungi | Ascomycota | Sordariomycetes | Sordariales | Lasiosphaeriaceae | Cladorrhinum | NA | 0.011 | CONFYM_0.7,CONFYM_1.4 | Saprotroph | Probable | |  |
|  | OTU368 | 0.038 | Fungi | Ascomycota | NA | NA | NA | NA | NA | 0.032 | CONFYM_1.4 | unclassified | na |  |  |
|  | OTU370 | 0.072 | Fungi | Ascomycota | NA | NA | NA | NA | NA | 0.028 | BIOORG_0.7,CONFYM_0.7 | unclassified | na |  |  |
|  | OTU379 | 0.080 | Fungi | NA | NA | NA | NA | NA | NA | 0.011 | BIODYN_1.4 | unclassified | na |  |  |
|  | OTU389 | 0.046 | Fungi | Basidiomycota | Agaricomycetes | Agaricales | Psathyrellaceae | NA | NA | 0.011 | CONFYM_1.4 | Saprotroph | Probable | |  |
|  | OTU4 | 2.901 | Fungi | NA | NA | NA | NA | NA | NA | 0.016 | BIODYN_0.7,BIODYN_1.4,BIOORG_0.7,BIOORG_1.4 | unclassified | na |  |  |
|  | OTU418 | 0.048 | Fungi | NA | NA | NA | NA | NA | NA | 0.013 | CONFYM_1.4 | unclassified | na |  |  |
|  | OTU420 | 0.031 | Fungi | Ascomycota | Dothideomycetes | Pleosporales | NA | NA | NA | 0.031 | CONFYM_0.7,CONFYM_1.4 | unclassified | na |  |  |
|  | OTU44 | 0.458 | Fungi | Ascomycota | Eurotiomycetes | Eurotiales | Aspergillaceae | NA | NA | 0.004 | BIODYN_0.7,BIOORG_0.7 | unclassified | na |  |  |
|  | OTU463 | 0.019 | Fungi | Ascomycota | Sordariomycetes | Sordariales | Lasiosphaeriaceae | Podospora | NA | 0.037 | CONFYM_0.7,CONFYM_1.4 | Saprotroph-Symbiotroph | Possible | |  |
|  | OTU49 | 0.347 | Fungi | NA | NA | NA | NA | NA | NA | 0.029 | BIOORG_0.7 | unclassified | na |  |  |
|  | OTU490 | 0.013 | Fungi | Ascomycota | NA | NA | NA | NA | NA | 0.035 | BIODYN_0.7,BIOORG_0.7 | unclassified | na |  |  |
|  | OTU510 | 0.028 | Fungi | Basidiomycota | Microbotryomycetes | Microbotryomycetes_ord_Incertae_sedis | Chrysozymaceae | NA | NA | 0.019 | CONFYM_1.4 | unclassified | na |  |  |
|  | OTU57 | 0.222 | Fungi | Ascomycota | Eurotiomycetes | Eurotiales | Aspergillaceae | Penicillium | NA | 0.047 | BIODYN_0.7,BIOORG_0.7 | Saprotroph | Highly Probable | |  |
|  | OTU580 | 0.020 | Fungi | Basidiomycota | Tremellomycetes | Trichosporonales | Trichosporonaceae | NA | NA | 0.027 | CONFYM_0.7,CONFYM_1.4 | unclassified | na |  |  |
|  | OTU59 | 0.333 | Fungi | Ascomycota | Sordariomycetes | Xylariales | NA | NA | NA | 0.002 | BIOORG_0.7,CONFYM_0.7 | unclassified | na |  |  |
|  | OTU595 | 0.021 | Fungi | Ascomycota | NA | NA | NA | NA | NA | 0.046 | BIODYN_1.4 | unclassified | na |  |  |
|  | OTU62 | 0.333 | Fungi | Ascomycota | Dothideomycetes | Pleosporales | NA | NA | NA | 0.044 | BIODYN_0.7,BIOORG_0.7,CONFYM_0.7 | unclassified | na |  |  |
|  | OTU64 | 0.748 | Fungi | Basidiomycota | Agaricomycetes | Cantharellales | Cantharellales_fam_Incertae_sedis | Minimedusa | Minimedusa_polyspora_SH1556024.08FU | 0.035 | BIODYN_0.7 | unclassified | na |  |  |
|  | OTU647 | 0.039 | Fungi | NA | NA | NA | NA | NA | NA | 0.002 | BIODYN_0.7,BIODYN_1.4 | unclassified | na |  |  |
|  | OTU665 | 0.017 | Fungi | Ascomycota | Eurotiomycetes | NA | NA | NA | NA | 0.011 | BIODYN_0.7,BIODYN_1.4 | unclassified | na |  |  |
|  | OTU681 | 0.021 | Fungi | NA | NA | NA | NA | NA | NA | 0.034 | CONFYM_1.4 | unclassified | na |  |  |
|  | OTU688 | 0.012 | Fungi | Ascomycota | Sordariomycetes | Sordariales | Sordariaceae | NA | NA | 0.032 | CONFYM_0.7,CONFYM_1.4 | Saprotroph | Probable | |  |
|  | OTU695 | 0.043 | Fungi | Basidiomycota | Agaricomycetes | NA | NA | NA | NA | 0.011 | BIODYN_1.4 | unclassified | na |  |  |
|  | OTU697 | 0.025 | Fungi | Mortierellomycota | Mortierellomycetes | Mortierellales | Mortierellaceae | Mortierella | NA | 0.020 | CONFYM_1.4 | Saprotroph-Symbiotroph | Probable | |  |
|  | OTU7 | 2.662 | Fungi | Ascomycota | Sordariomycetes | Hypocreales | NA | NA | NA | 0.011 | CONFYM_1.4 | unclassified | na |  |  |
|  | OTU70 | 0.405 | Fungi | Ascomycota | Pezizomycetes | Pezizales | Ascobolaceae | NA | NA | 0.002 | CONFYM_0.7,CONFYM_1.4 | Saprotroph | Probable | |  |
|  | OTU77 | 0.298 | Fungi | Ascomycota | Sordariomycetes | Xylariales | NA | NA | NA | 0.022 | BIODYN_0.7,BIODYN_1.4,BIOORG_0.7,BIOORG_1.4 | unclassified | na |  |  |
|  | OTU78 | 0.340 | Fungi | Ascomycota | NA | NA | NA | NA | NA | 0.044 | BIOORG_0.7 | unclassified | na |  |  |
|  | OTU8 | 4.403 | Fungi | Mortierellomycota | Mortierellomycetes | Mortierellales | Mortierellaceae | NA | NA | 0.009 | BIODYN_0.7,BIODYN_1.4,BIOORG_1.4,CONFYM_1.4 | Saprotroph-Symbiotroph | Possible | |  |
|  | OTU81 | 0.334 | Fungi | Ascomycota | Sordariomycetes | Hypocreales | Clavicipitaceae | NA | NA | 0.019 | BIOORG_0.7,CONFYM_0.7,CONFYM_1.4 | Pathotroph-Symbiotroph | Possible | |  |
|  | OTU82 | 0.216 | Fungi | Ascomycota | Sordariomycetes | Sordariales | Chaetomiaceae | NA | NA | 0.031 | BIODYN_0.7,BIOORG_0.7,CONFYM_0.7 | unclassified | na |  |  |
|  | OTU836 | 0.011 | Fungi | Ascomycota | Sordariomycetes | Glomerellales | Plectosphaerellaceae | NA | NA | 0.026 | BIOORG_0.7,BIOORG_1.4 | Pathotroph-Saprotroph-Symbiotroph | Possible | |  |
|  | OTU85 | 0.289 | Fungi | Ascomycota | Sordariomycetes | Coniochaetales | Coniochaetaceae | Lecythophora | NA | 0.039 | BIODYN_0.7,BIODYN_1.4,BIOORG_1.4,CONFYM_1.4 | Symbiotroph | Highly Probable | |  |
|  | OTU86 | 0.684 | Fungi | Ascomycota | Sordariomycetes | Sordariales | Lasiosphaeriaceae | NA | NA | 0.011 | BIOORG_0.7 | Saprotroph | Probable | |  |
|  | OTU89 | 0.730 | Fungi | Mortierellomycota | Mortierellomycetes | Mortierellales | Mortierellaceae | Mortierella | Mortierella_alpina_SH1650285.08FU | 0.004 | BIODYN_0.7,BIODYN_1.4,BIOORG_1.4 | Saprotroph-Symbiotroph | Probable | |  |
|  | OTU92 | 0.283 | Fungi | Ascomycota | Sordariomycetes | NA | NA | NA | NA | 0.011 | BIODYN_1.4 | unclassified | na |  |  |
|  | OTU945 | 0.011 | Fungi | NA | NA | NA | NA | NA | NA | 0.032 | BIODYN_0.7,BIOORG_0.7 | unclassified | na |  |  |
|  | OTU96 | 0.262 | Fungi | Ascomycota | NA | NA | NA | NA | NA | 0.011 | BIOORG_0.7,CONFYM_0.7 | unclassified | na |  |  |
|  | OTU990 | 0.083 | Fungi | Ascomycota | Pezizomycetes | Pezizales | Pyronemataceae | NA | NA | 0.011 | CONFYM_1.4 | Saprotroph-Symbiotroph | Possible | |  |
