# Supplementary material for: Soil microbial communities are sensitive to differences in fertilization intensity in organic and conventional farming systems
Source: FEMS Microbiol Ecol. 2023 May 9;99(6):fiad046. doi: 10.1093/femsec/fiad046 (PMC10236208; doi:10.1093/femsec/fiad046)
Supplement: fiad046_Supplemental_Files [file fiad046_supplemental_files.zip › Supplemtary_Figure_1.pdf]

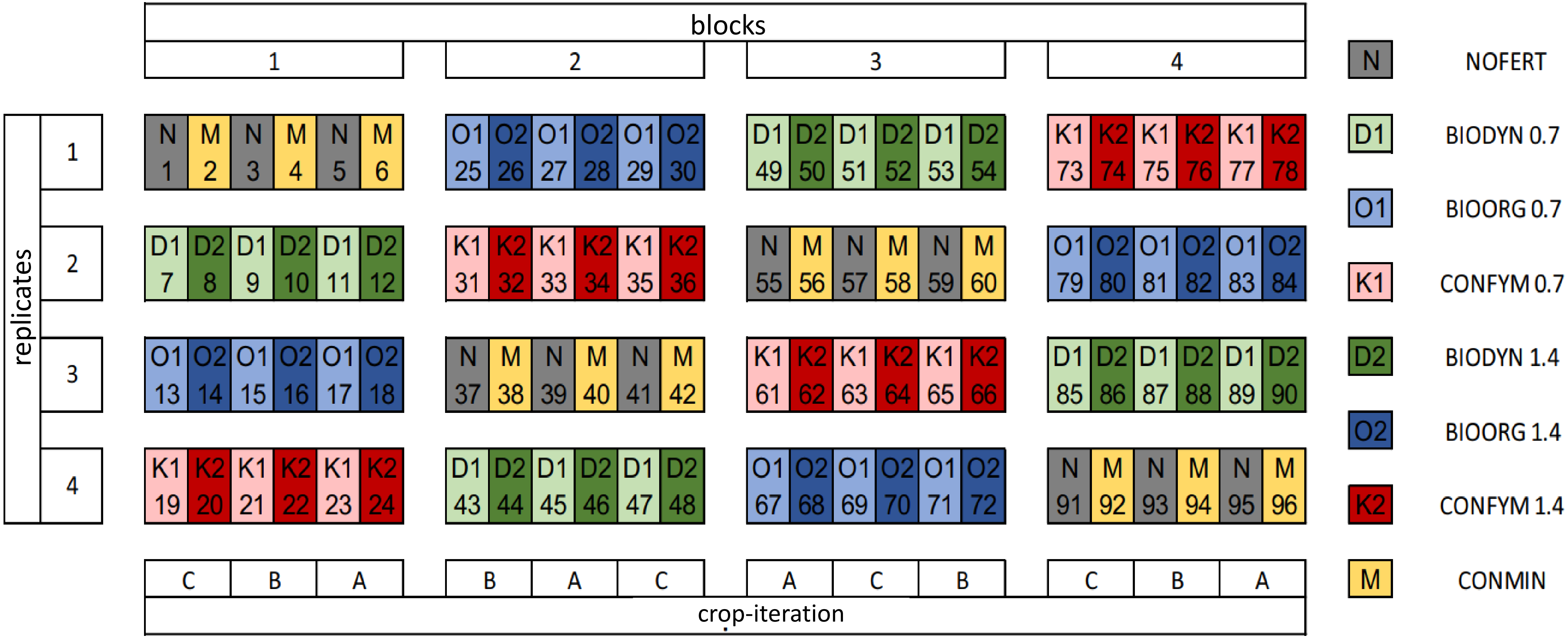
blocks
1
2
3
4
replicates
1
N 1 M 2 N 3 M 4 N 5 M 6
O1 25 O2 26 O1 27 O2 28 O1 29 O2 30
D1 49 D2 50 D1 51 D2 52 D1 53 D2 54
K1 73 K2 74 K1 75 K2 76 K1 77 K2 78
2
D1 7 D2 8 D1 9 D2 10 D1 11 D2 12
K1 31 K2 32 K1 33 K2 34 K1 35 K2 36
N 55 M 56 N 57 M 58 N 59 M 60
O1 79 O2 80 O1 81 O2 82 O1 83 O2 84
3
O1 13 O2 14 O1 15 O2 16 O1 17 O2 18
N 37 M 38 N 39 M 40 N 41 M 42
K1 61 K2 62 K1 63 K2 64 K1 65 K2 66
D1 85 D2 86 D1 87 D2 88 D1 89 D2 90
4
K1 19 K2 20 K1 21 K2 22 K1 23 K2 24
D1 43 D2 44 D1 45 D2 46 D1 47 D2 48
O1 67 O2 68 O1 69 O2 70 O1 71 O2 72
N 91 M 92 N 93 M 94 N 95 M 96
C B A
B A C
A C B
C B A
crop-iteration
N NOFERT
D1 BIODYN 0.7
O1 BIOORG 0.7
K1 CONFYM 0.7
D2 BIODYN 1.4
O2 BIOORG 1.4
K2 CONFYM 1.4
M CONMIN
