# Supplementary figures and images for: Soil microbial communities are sensitive to differences in fertilization intensity in organic and conventional farming systems
Source: FEMS Microbiol Ecol. 2023 May 9;99(6):fiad046. doi: 10.1093/femsec/fiad046 (PMC10236208; doi:10.1093/femsec/fiad046)

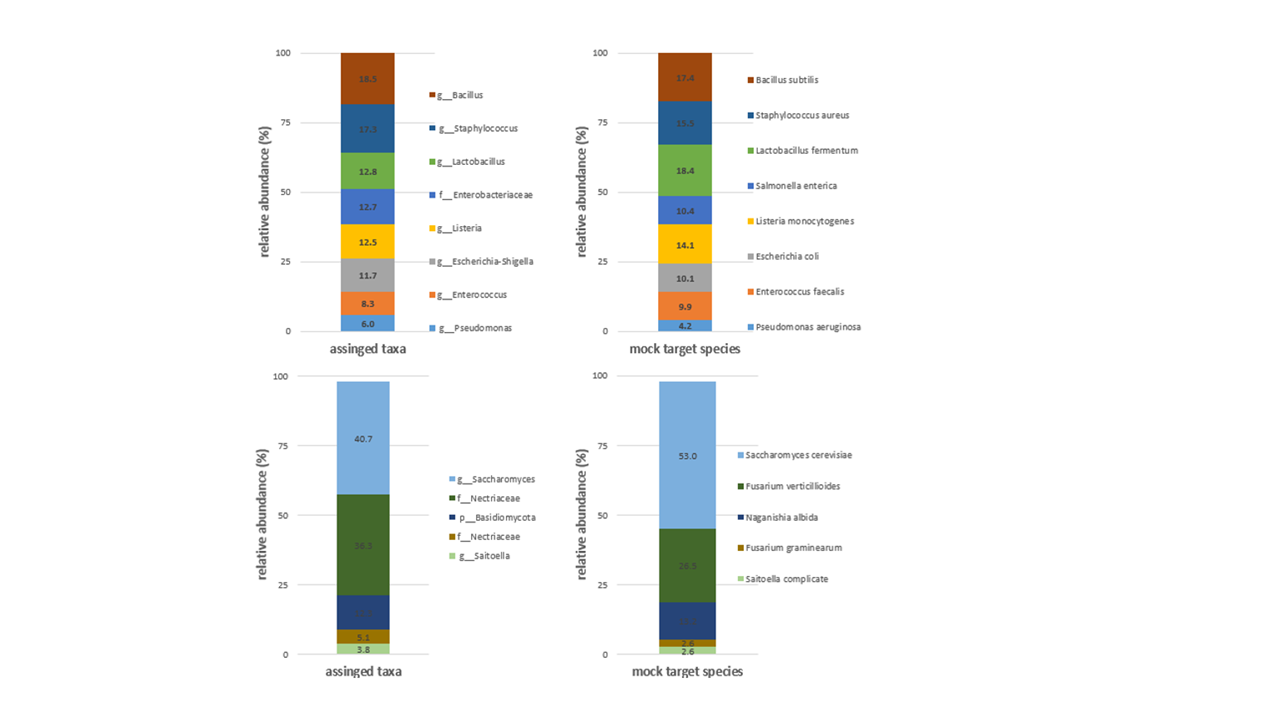

Supplement: fiad046_Supplemental_Files [file fiad046_supplemental_files.zip › Supplementary_Figure2.png]

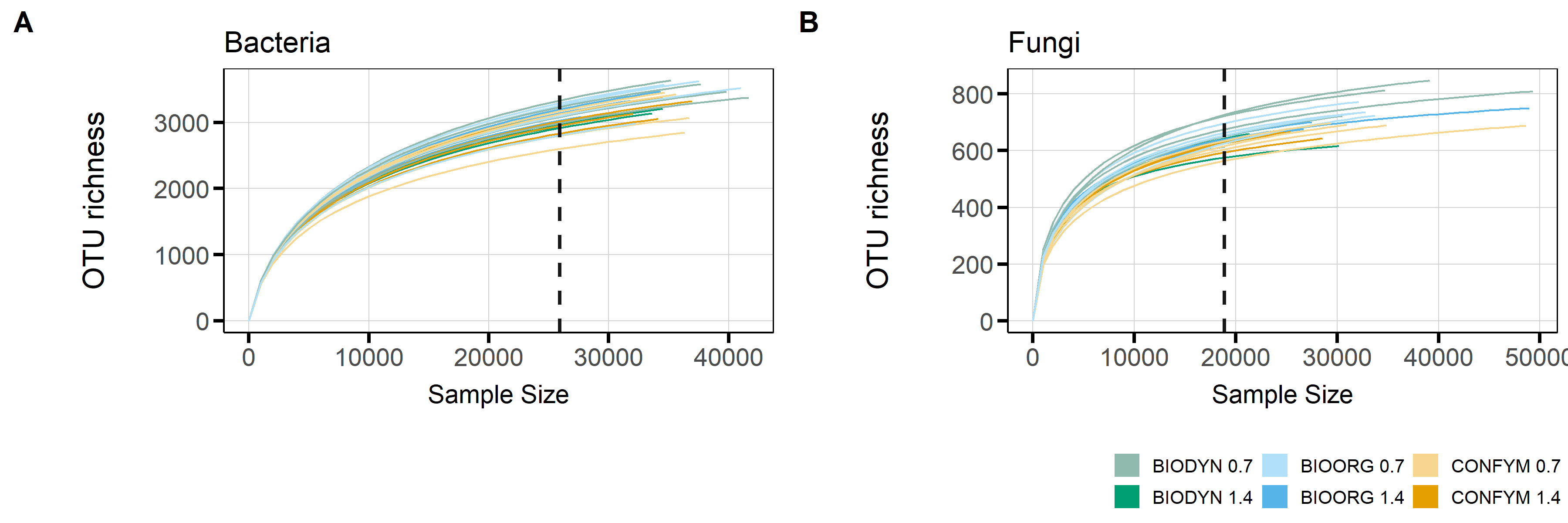

Supplement: fiad046_Supplemental_Files [file fiad046_supplemental_files.zip › Supplementary_Figure3.png]

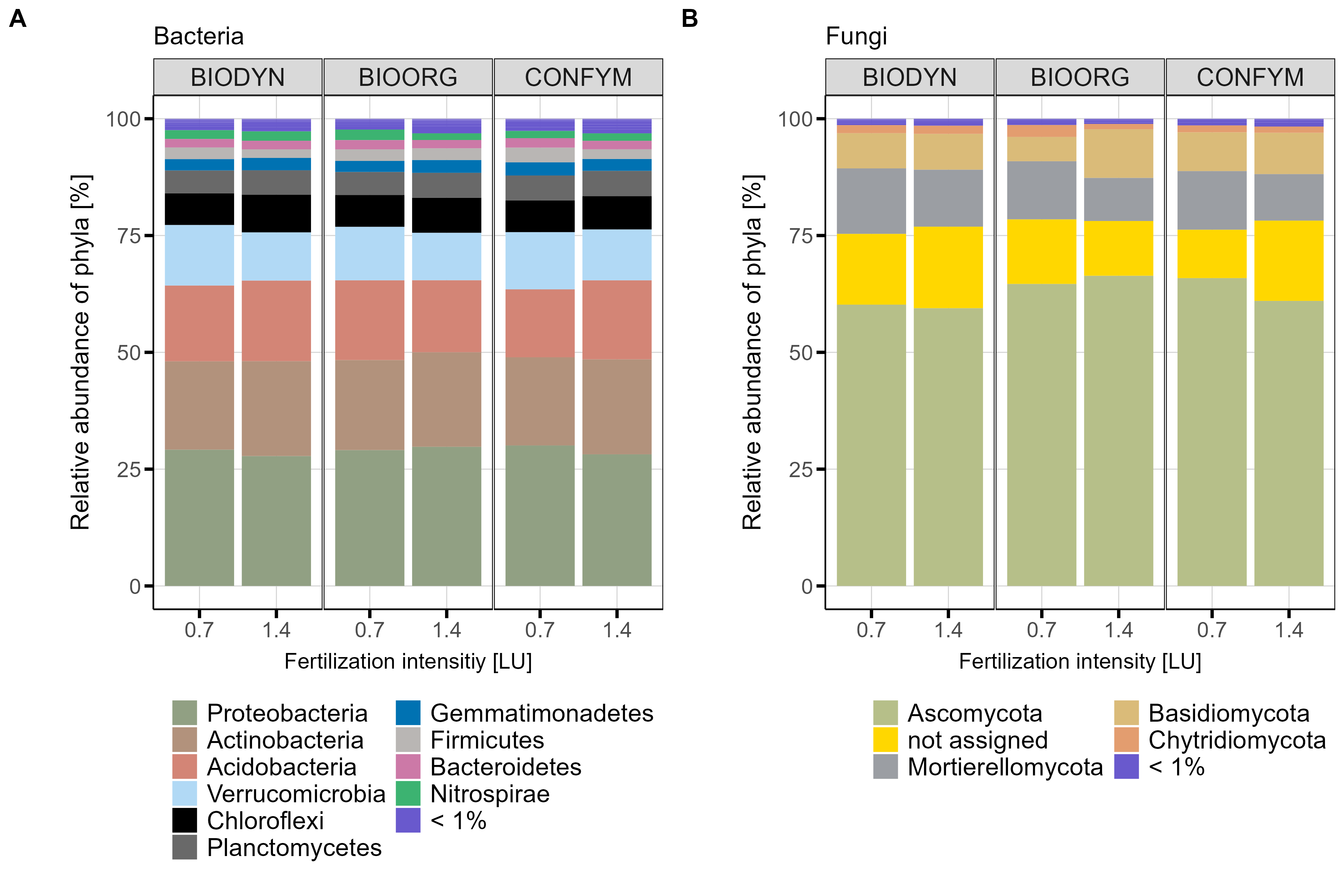

Supplement: fiad046_Supplemental_Files [file fiad046_supplemental_files.zip › Supplementary_Figure_4.png]

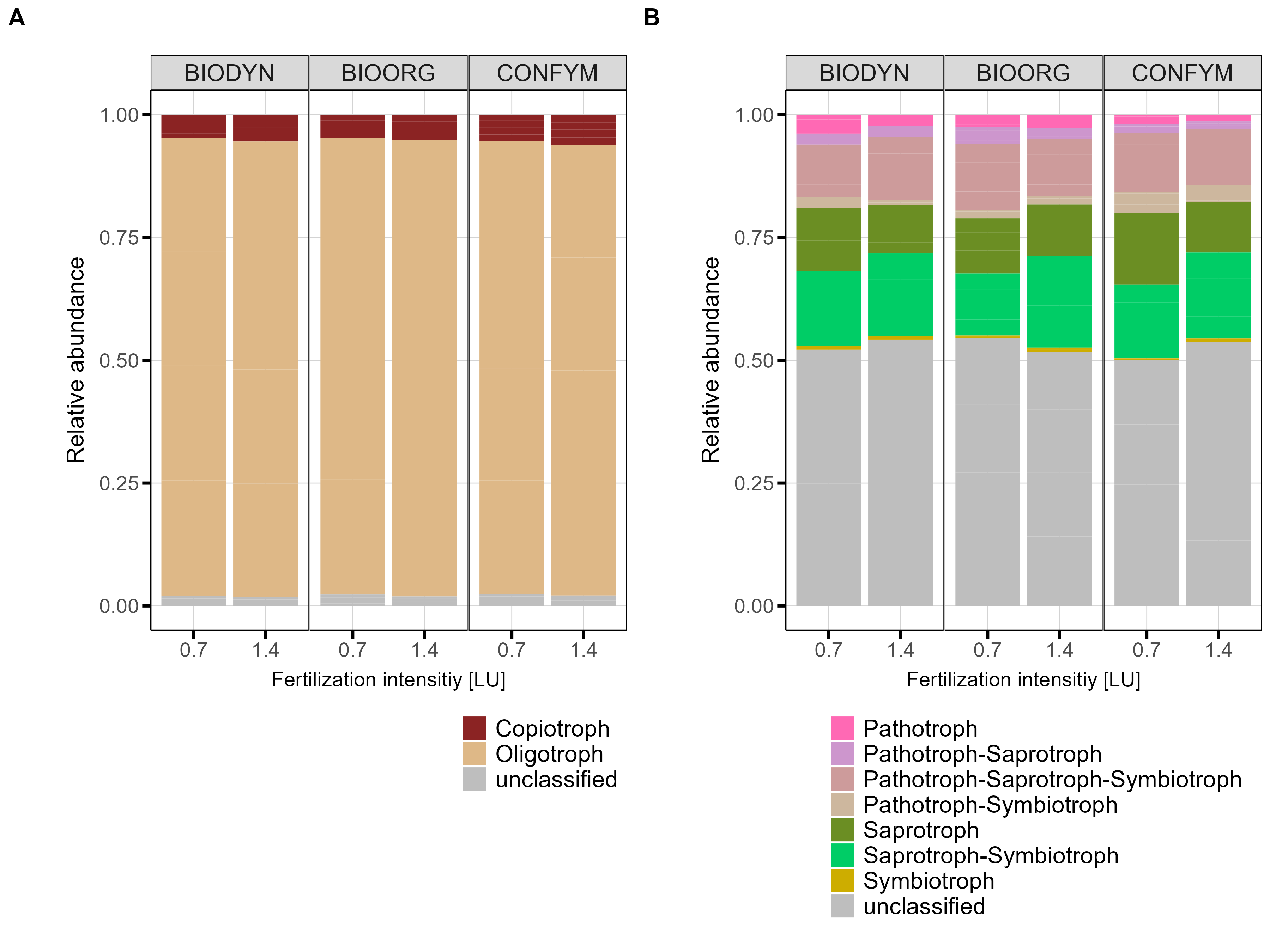

Supplement: fiad046_Supplemental_Files [file fiad046_supplemental_files.zip › Supplementary_Figure_5.png]
